# Designed Multifunctional Peptides for Intracellular Targets

**DOI:** 10.3390/antibiotics11091196

**Published:** 2022-09-03

**Authors:** Davor Juretić

**Affiliations:** 1 Mediterranean Institute for Life Sciences, 21000 Split, Croatia; djuretic@medils.org; 2 Faculty of Science, University of Split, 21000 Split, Croatia; juretic@pmfst.hr

**Keywords:** amphipathic peptides, multifunctional, design, penetratins, antimicrobial, antiviral, anticancer, anti-inflammatory, cell-penetrating, non-toxic

## Abstract

Nature’s way for bioactive peptides is to provide them with several related functions and the ability to cooperate in performing their job. Natural cell-penetrating peptides (CPP), such as penetratins, inspired the design of multifunctional constructs with CPP ability. This review focuses on known and novel peptides that can easily reach intracellular targets with little or no toxicity to mammalian cells. All peptide candidates were evaluated and ranked according to the predictions of low toxicity to mammalian cells and broad-spectrum activity. The final set of the 20 best peptide candidates contains the peptides optimized for cell-penetrating, antimicrobial, anticancer, antiviral, antifungal, and anti-inflammatory activity. Their predicted features are intrinsic disorder and the ability to acquire an amphipathic structure upon contact with membranes or nucleic acids. In conclusion, the review argues for exploring wide-spectrum multifunctionality for novel nontoxic hybrids with cell-penetrating peptides.

## 1. Introduction

Bioactive peptides are all around us, including host defense peptides (HFD) in our bodies. We can regard them as templates developed by natural evolution that are lead compounds for creating commercial products or drugs. Various chemical modifications are employed to increase their stability for different applications. Bioactive peptides are often multifunctional. Some are hidden within proteins and liberated to perform their functions only when needed. Others can be designed in silico by combining several shorter peptides. In any case, there is a fast-growing field of design and applications for peptides that may have multifaceted performance. Such candidate therapeutics may help treat complex diseases often associated with opportunistic infections. Dual antibacterial and anticancer activity has been frequently observed [1,2,3,4,5,6]. For instance, wide-range antibacterial peptide aurein 1.2 exhibits high activity against 52 cancer cell lines [7]. Another nontoxic antimicrobial peptide, buforin IIb, is active against 60 human tumor cell lines [8]. The bimodal function can encompass antimicrobial and anti-inflammatory activity [9,10,11]. Hilchie et al. [9] mention 18 biological activities of cationic host defense peptides and their synthetic derivatives. In their 2019 review [12], Hilchie et al. stressed that “cationic amphipathic peptides may exhibit any combination of antimicrobial, anticancer, or immune-modulatory properties”.

Regarding antimicrobial performance, antifungal and antiviral activity are of particular interest due to difficulties in the development of safe, low molecular weight antibiotics against such targets [13,14,15,16,17]. The penetration inside cells also belongs to the coveted multifunctional property, firstly for the ability of cell-penetrating peptides (CPP) to interact with the cellular membrane in a non-invasive manner [18,19], and secondly for acting on hard-to-reach intracellular targets [20,21].

Current algorithms for predicting the activity of multifunctional peptides have limited accuracy. However, they are still helpful indicators of which natural peptides or in silico constructs are promising for much more expensive verifications in vitro and in vivo. A plethora of user-friendly servers has appeared during recent years for sequence-based prediction of cell-penetrating (CPP), antimicrobial (AMP), anticancer (ACP), antiviral (AVP), antifungal (AFP), and anti-inflammatory (AIP) peptides [22,23,24,25,26,27,28,29,30,31,32,33,34,35]. An older server by Hwang et al. [36] can be used to predict DNA binding. A valuable feature is when servers allow for designing novel peptides with improved function [35] or decreased toxicity [37]. The goal of combining all six activities (CPP, AMP, ACP, AVP, AFP, and AIP) in a single peptide construct is possible, but two caveats should be considered. We do not want to invest time and money into examining strongly toxic peptides. Fortunately, in silico prediction by dedicated servers for toxicity [37,38,39] and hemolytic activity [40] can be used to prune designed candidates with high predicted hemolytic activity or toxicity to healthy human cells. Secondly, all predictions are questionable in the absence of experimental validation. Hence, whenever possible, we must compare predictions with observations to obtain insight into the reliability of employed “in silico” expectations.

We shall describe in this review several classes of peptides that have confirmed or predicted high multifunctional potential. Our approach is to start with some natural or artificial peptides with proven cell-transduction efficiency. It is the parent peptide for in silico exploration on how it can be modified or fused to other bioactive peptides for acquiring multifunctional activity without losing its cell-penetrating ability. Such peptides have a better chance of reaching intracellular pathogens that are difficult to eradicate with conventional antibiotics.

Regarding predictions, there are several additional caveats to using publicly accessible web servers for predicting sequence-based functionality for a peptide. The most important one is reproducibility. Free assistance to the scientific community via such web servers is never cost-free for those who maintain them. Suppose larger organizations up to the state or international level are not involved in maintaining long-term reproducibility. In that case, the half-life of servers for scientific calculations is measured in years, not decades. The most severe reproducibility problem is when the server’s output (score) is different for each submission of an identical peptide. That may happen when recent algorithms are still riddled with bugs; although, their link is in the public domain and the description is published in a high-impact journal. The case example is the ToxIBTL server for predicting peptide toxicity [41].

Different artificial intelligence algorithms are becoming ever more popular in constructing predictive algorithms. However, most suffer from well-known weaknesses. They are essentially black boxes containing some rules learned during the training procedure. There is no easy way to discover and formulate these rules, however useful they may be in raising the prediction accuracy. Overly intensive training does not help either because it can decrease the performance when the AI algorithm is presented with the testing dataset, which differs in some properties from the training dataset.

When large enough datasets of non-redundant and non-homologous peptides are collected, one can separate the training and testing datasets by choosing some compromise for the cut-off in similarity among these datasets. It is an excellent practice when several benchmarking datasets are used for testing. However, the proper training procedure should be such that testing datasets are never examined during the training procedure. Tests with the benchmark datasets should be done only once. Frequent jackknife tests of the training dataset amount to additional training procedures and should be avoided if possible. It may not be possible when different descriptors are tested as well.

The fourth caveat is connected to the choice of features or descriptors. It is subjective and usually limited to overly simple ideas about what is essential for peptides’ activity. Atomic composition, amino acid composition, dipeptides composition, charges, and other amino acid features (hydrophobicity) completely neglect the sequence order of amino acid residues in a peptide, sequence profile of hydrophobicity and hydrophobic moments, dipole moments, and many other structure-associated physicochemical features. These are features and descriptors we described in our publications when we were constructing descriptors for predicting selectivity and a membrane-induced increase in helical conformation [42,43,44,45,46]. Recently developed AI algorithms, which we mentioned in Methods, incorporate interpretable features and in-depth analysis of peptides’ biophysical and biochemical properties. We have used them on many occasions during the past several years. There were only occasional short service disruptions for some of them, probably due to maintenance. Our last accession was on 7 August 2022.

We shall firstly examine in this work the multitude of natural penetratin analogs with special attention to those of ancient origin. Secondly, we shall use the hybrid constructs with penetratin analogs and optimized penetratin to find promising lead compounds for strong multifunctional activity. Thirdly, novel peptide conjugates for intracellular targets will be proposed too. Next, shorter CPPs unrelated to penetratin, either known or novel, will be examined regarding predicted multifunctional activities when conjugated to peptides with verified activity for promising broad-spectrum applications.

Conclusions will gather the best compromise for all peptide constructs among strongly predicted six multifunctional activities (CPP, AMP, ACP, AVP, AFP, and AIP) and low toxicity estimates in the hope of future experimental verifications and appropriate chemical modifications for various applications. The class of highly charged temporin analogs fused to short CPP ended up as 50% of the 20 best peptides that have promising therapeutic potential. They are not overly expensive for synthesis, with a length ranging from 22 to 31 amino acid residues.

## 2. Sequence-Based Servers for Predicting Peptide Activity and Proposed Ranking Methods

The choice of online available predictive algorithms is according to (a) their online persistence, (b) the usage simplicity when peptide sequence is submitted, and (c) claimed accuracy. The last requirement (accuracy) is challenging to estimate independently from the authors’ claims. Prediction results are commented on in the paper when they indicate some algorithm shortcomings.

The **MLCPP** server, www.thegleelab.org/MLCPP/ (accessed on 7 August 2022) by Manavalan et al. [22], is used to predict peptide cell-penetrating probability and uptake efficiency. We also consulted the **C2Pred** server by Tang et al. [23] (http://lin-group.cn/server/C2Pred, (accessed on 7 August 2022)) for the CPP probability.

The **DP-Bind** server http://lcg.rit.albany.edu/dp-bind/ (accessed on 7 August 2022) by Hwang et al. [36] is used for sequence-based prediction of DNA-binding residues in DNA-binding proteins and peptides. In some cases, the **dSPRINT** server http://protdomain.princeton.edu/dsprint, (accessed on 7 August 2022)) by Etzion-Fuchs et al. [47] provided the confirmation of the DNA-binding preference for sequence domains.

The antimicrobial peptide probability for a query peptide is found by applying the Support Vector Machine (SVM) algorithm from the **CAMP_R3_** web server http://www.camp.bicnirrh.res.in/predict (accessed on 7 August 2022) [24]. We also used the **AmpGram** server (http://biongram.biotech.uni.wroc.pl/AmpGram/ (accessed on 7 August 2022) [25]) to identify antimicrobial peptides.

Two web servers are used to predict the peptide’s anticancer activity. These are the **ACPred** server http://codes.bio/acpred/ (accessed on 7 August 2022) [26] and the **mACPred** server http://thegleelab.org/mACPpred/ (accessed on 7 August 2022) by Boopathi et al. [27].

Three web servers are used to predict the peptide’s antiviral activity. These are the **ENNAVIA** server https://research.timmons.eu/ennavia (accessed on 7 August 2022) by Timmons and Hewage [28], the **FIRM-AVP** server https://msc-viz.emsl.pnnl.gov/AVPR/ (accessed on 7 August 2022) by Chowdhury et al. [29], and the **Meta-iAVP** server http://codes.bio/meta-iavp/ (accessed on 7 August 2022) by Schaduangrat et al. [30].

The **iAMPpred** web server http://cabgrid.res.in:8080/amppred/server.php (accessed on 7 August 2022) of Meher et al. [31] gives predictions for antibacterial, antiviral, and antifungal activity, but we reported only the last one. We also used the **AntiFungal** server of Zhang et al. [32] (https://www.chemoinfolab.com/antifungal/, (accessed on 7 August 2022)) to predict the antifungal activity.

For the prediction of anti-inflammatory activity, we used the **AIPpred** server (http://www.thegleelab.org/AIPpred/ (accessed on 7 August 2022) [33]), the **PreAIP** server (http://kurata14.bio.kyutech.ac.jp/PreAIP/ (accessed on 7 August 2022) [34]), and the scoring output of the **AntiInflam** server (http://metagenomics.iiserb.ac.in/antiinflam/ (accessed on 7 August 2022) [35]) when it predicts the anti-inflammatory activity. We used the AntiInfam server to design peptides with a better anti-inflammatory score.

Two different methods estimated peptide toxicity. Firstly, the probability that the peptide has hemolytic activity was assessed using the **HAPPENN** server https://research.timmons.eu/happenn (accessed on 7 August 2022) by Timmons et al. [40]. Secondly, the peptide toxicity was predicted by the **ToxinPred** server https://webs.iiitd.edu.in/raghava/toxinpred/ (accessed on 7 August 2022) [37,38,39]. We used the server modules for batch submission and designing peptides with decreased toxicity. To verify peptide toxicity class (toxic or nontoxic), a more recent **ToxIBTL** server http://server.wei-group.net/ToxIBTL (accessed on 7 August 2022) [41] was also employed. Besides toxicity class, that server’s output contains an irreproducible and meaningless score because the user is given a different score for an identical peptide in each submission.

We employed older reliable servers, **SPLIT 3.5** [42] and **SPLIT 4.0** [43], for predicting the sequence profile of hydrophobicities, optimal hydrophobic moments, and membrane preference for amphipathic and membrane-associated segments: http://split.djpept.com/split/ (accessed on 7 August 2022) and http://split.djpept.com/split/4/ (accessed on 7 August 2022). Our **Mutator** tool [46] served to design anuran-like peptide antibiotics with a predicted high selectivity index: http://mutator.djpept.com/ (accessed on 7 August 2022) or http://splitbioinf.pmfst.hr/mutator/ (accessed on 7 August 2022).

For each of the considered peptides, we presented predicted results in Table 1, Table 2, Table 3, Table 4 and Table 5. The summary Table 6 for ranking the best peptide constructs presents only mean scores for each of the predicted activities. The mean score for anti-inflammatory activity can be higher than 1.0 because the AntiInflam server reports the score for the AIP activity that can be higher than 1.0. The arithmetic average of mean CPP, AMP, ACP, AVP, AFP, and AIP scores served to rank all peptides regardless of their toxicity to healthy human cells. We then introduced the reward for predicted low toxicity and hemolytic activity to obtain the overall ranking for all nontoxic multifunctional constructs. The reward score is calculated as a negative mean of toxicity score (negative) by the ToxinPred server and the HAPPENN server output (positive). Mean scores for six activities and the reward score are then averaged to obtain the overall score. It ranges from 0.873 to 0.927 for the 20 best peptides, while the reward score ranges from 0.346 to 0.867.

The overall score ranking is highly dependent on estimated toxicity. Peptide toxicity is usually firstly examined as hemolytic potency. Minimizing hemolytic activity can improve the therapeutic potential of peptides. The HAPPENN server [40] employs the threshold value of 0.5 to distinguish hemolytic from non-hemolytic peptides. Its valuable feature is distinguishing C-terminal amidated from non-amidated peptides. Amidated peptides are more active antimicrobials but can be associated with increased hemolytic activity. Magainin-2 in its C-terminal amidated form is the best-known antimicrobial peptide. More than 500 μM concentration of MG2 is needed to cause 50% hemolysis. Its hemolytic probability is 0.83 (see Table 5, peptide 6 for the HAPPENN output). Therefore, a peptide with a probability for hemolytic activity between 0.50 and 0.83 or less can still be a good candidate for synthesis, purification, and testing.

## 3. Under-Appreciated Versatility of Penetratins

### 3.1. The Evolutionary Depth of Homeobox Domains and Penetratin-like Cryptides in the Animalia Kingdom

Natural DNA-binding peptides can be the inspiration for designing cell-penetrating peptides (CPP) with DNA-binding and other multifunctional activities. We shall first explore this idea for the penetratin-like peptides. Le Roux et al. published in 1993 [48], the primary structure of 35 amino acid long cryptide L(322)TRRRRIEIAHALCLTE**RQIKIWFQNRRMKWKK**EN(356) rich in arginines from the homeodomain of the Drosophila melanogaster (fruit fly) protein Antennapedia (pAntp). The highlighted sequence (with bold font residues) was named the penetratin peptide. Remarkably, that 16-residues long cryptide (hidden peptide) from homeodomain proteins connected fruit flies to humans (Table 1). One can speculate that DNA-binding and cell-penetrating functions are related and equally ancient for penetratin analogs found in homeobox-like proteins (Table 1 and Table 2). More to the point, membrane activity, cell-penetrating ability, antimicrobial potency, and anticancer activity are also related to the highly cationic and moderately amphipathic structure of the penetratin and its natural or synthetic analogs [49,50,51,52,53,54,55,56,57,58].

Identical hexadecapeptide penetratin analog is present in Drosophila O18381, mouse P63015, and human P26367 Pax-6 parent proteins. It is the arginine-rich AR**I**QV**WF**S**NRR**A**KW**RR sequence (residues identical to *Drosophila* pAntp penetratin are in a bold font). We can estimate its evolutionary depth by performing the peptide search for that arginine-rich sequence in the UniProt database. There are about two thousand hits for invertebrate and vertebrate animals, most associated with the Pax-6 annotation. The *Pax-6* gene is a master control gene responsible for developing photodetection and eye morphogenesis in flies, mice, and humans. Walter Gehring and his co-authors postulated that the strikingly diverse eyes found in the most primitive to the most advanced animals derived from an ancestral eye and ancestral organ selector genes [59,60,61,62,63]. Pax and Pax-like genes coding for penetratin analogs were found not only in flatworms, insects, and mammals but also in sponges lacking a nervous system [64,65,66].

Corresponding proteins are transcription factors containing two to three domains with three α-helices. The first two domains belong to the defining Pax signature of the 128-amino acid DNA-binding paired domain [67]. The third DNA-binding domain with three helices is the 60-amino acid homeobox domain. Binding to DNA as homodimers or heterodimers is often essential for the transcriptional activity of homeobox-containing proteins [68]. An unresolved question is the functional importance of penetratin analogs found in a homeobox-like sequence of the simplest and most ancient animals devoid of organs. Another underexplored question regards the possible toxicity of natural or designed penetratin analogs. When substituted amino acids change peptide–DNA or parent protein–DNA interaction, the results can be either beneficial or harmful in vivo. Disease-causing mutations in the human Pax3 gene belong to the latter examples.

From the UniProt entry P23760 the homeobox sequence is Q(219)RRSRTTFTAEQLEEL(234)ERAF(238)ERTHYPDIYTREELAQRAKLTEARVQV(265)W(266)FSNR(270)R(271)AR(273)WRKQA(278) for human Pax3 (we underlined helices α1, α2, and α3). The substitution of residues V(265), W(266), R(270), R(271), and R(273) from recognition helix α3 with, respectively, F, C, C, C, and K, may result in the Waardenburg syndrome (WS1) with impaired hearing and other disorders. Presumably, Phe (F) and Cys (C) cannot maintain crucial DNA–homeodomain interactions provided by V(265), W(266), and R(271). Substitutions P for L(234) and S for F(238) are also causing WS1 syndrome probably by destabilizing the hydrophobic interactions for the homeodomain fold (see Birrane et al., 2009 paper [69], where L(16) and F(20) correspond to L(234) and F(238)). Birrane et al. [69] concluded that Pax3 has no DNA-interacting residue in its first homeodomain helix (α1). It has one DNA-interacting residue in its second helix (α2) and eight such residues in its third DNA-recognition helix (α3). Other authors also concluded that the penetratin-like helix α3 has the strongest contact with the major DNA groove [70,71].

We restricted Table 1 examples of metazoan penetratins to phylums Chordata (Mammalia class), Tunicata (subphylum, Ascidiacea class, which includes sea squirts), Antrophod (Insecta class), Annelida (Polychaeta class worm), Cnidaria (Anthozoa class, including stony corals), Ctenophora (Tentaculata class, which includes comb jellies), Porifera (Despongiae class), and Placozoa (*T. adhaerens*). In all subkingdoms of Animalia, we can easily find those penetratin analogs that are essential motifs in transcription factors regulating the development.

Given examples from Table 1, let us elaborate on the evolutionary depth of the conserved role for Pax, Pax-like genes, homeotic genes, and associated penetratin-like DNA-binding motifs. It is not only penetratin-like peptides from animals without eyes, eye spots, and neurons (Table 1 examples for Porifera and Placozoa). Surprisingly, such peptides are also present in fungi, yeasts, bacteria, Archaea, and viruses. In his 2013 review, Peter Holland observed that homeotic genes were not found in Archaea or bacteria [72]. However, additional Archaea and bacterial genomes have been decoded during the past decade. The last nine rows from Table 1 illustrate that homeobox domains and penetratin analogs can be found as cryptides among proteins from prokaryotic cells and viruses. The bacterial origin is more likely than the Archaea origin for a recognizable homeodomain with the helix-loop-helix-turn-helix motif. Only marginal similarity to pAntp or human Pax-6 penetratin is found for natural penetratin analogs from Archaea because at least 50% of the residues from these hexadecapeptides are different. Recent whole-genome decoding of giant viruses also revealed putative homeodomains and penetratin analogs [73,74]. The conserved motif WFXNRR is shared among all kingdoms of life, but it is too short to find significant similarities. In any case, prokaryotes and viruses also use regulatory transcription factors, and some of them may have been the progenitors of homeotic proteins in eukaryotes.

Ed Lewis, the first expert on homeotic genes, quipped in a letter to Walter Gehring: “Dear Walter, you made the homeobox our flying carpet.” The penetratin analog segments are our time-machine part of the “flying carpet“ for reaching the distant past of Life development. Let us show several examples to support that claim. We used our PROSITE motifs, BLASTP, and UniProt searches to investigate the evolutionary roots. That is the origin of some of the cited penetratin analogs (see Table 1 and Table 2).

Example 1: Human penetratin-like sequences

There are more than 500 human homeotic proteins. Some human proteins contain two homeobox domains and two different penetratin-like peptides (see some examples at UniProt links O43812, Q96PT3, A6NLW8, and P0CJ85). Human Zink finger homeobox protein 3 has four homeobox domains in its long sequence of 3703 residues (see Q15911) with four associated penetratins, which are, however, of low similarity to pAntp penetratin.

Example 2: Nematodes, cnidarians, and tunicates

Previously mentioned arginine-rich analog ARIQVWFSNRRAKWRR is present in the Vab-3 transcription factor G5EDS1 from the worm *Caenorhabditis elegans*. The worm does not have eyespots, much less fully developed eyes. Since it lives underground or inside rotting fruits, it does not require image-forming eyes, however primitive. Still, the worm has consistently expressed the Pax6 gene [66], which must be somehow involved in developing its miniature brain. *C. elegans* uses rhodopsin-like sensory receptor protein Q10042 annotated with a G protein-coupled receptor activity, but molecular details of its function are unknown. Color-perceiving systems without eyes and without ”seeing“ color may exist. The *C. elegans* animal model is probably the best for discovering neural circuits and previously unrecognized proteins that have evolved to capture light and react to rich information within the light spectrum. Its nervous system consists of only 302 neurons and performs miracles of sensing mechanical forces, chemicals, temperature, humidity, and electromagnetic fields. The Vab-3 involvement (if any) in *C. elegans* neural circuits for eyeless light detection is still the subject of active research.

The same arginine-rich sequence is present in the *Nematostella vectensis* (sea anemone) PaxC homeodomain from the transcription factor Q5IGV4. That cnidarian has a variable number of neurons (several hundred at most [75]) in decentralized nerve nets and poorly understood eyeless photodetection [76]). Another cnidarian, the *Acropora millepora* stony coral, can tune spawning behavior with the phases of the moonlight [77]. It is unknown whether the penetratin analog ARIQVWFSNRRAKWRK from Q5IGV4 protein, with a conservative Arg to Lys substitution, plays a role in light sensing by coral larva or not. It would not be surprising that more ancient eyeless vision needed penetratin analogs for its development. The arginine-rich hexadecapeptide connects worms, corals, and starlet sea anemones to insects and mammals. Its sequence can be as good a, if not a better, vehicle than pAntp penetratin for trans-membrane transport.

Tunicates are the sister group to vertebrates. The *Ciona intestinalis* larva (sea squirt tunicate) has the smallest brain of any chordate, with only 231 neurons [78]. Still, it needs the transcription factor protein NP_001071798.1 containing the penetratin-like ARVQVWFSNRRAKWRR sequence. Larva’s simple eye-spot ocellus has a pigment cell and vertebrate type ciliary opsin Ci-opsin1 [79], showing significant homology to vertebrate rhodopsins [80]. The retinal chromophore, Ci-opsin1, ocellus, and homeobox-containing transcription factors are the connection to the evolution of complex vertebrate eyes.

Example 3: Placozoans

Placozoans are the simplest animals in the evolutionary tree of Metazoa. The expression of homeobox-containing proteins has been confirmed in *Trichoplax adhaerens* and other placozoans [81,82,83]. *T. adhaerens* express genes encoding for proteins implicated in morphogenesis [84], innate immunity [85,86,87,88,89,90], and motility [91]. Moving and sensing are possible without brain cells but not without specialized proteins. The ARVQVWFSNRRAKWRR penetratin analog from the *T. adhaerens* ACH57174.1 Pax-3-like protein is different from corresponding human analogs only in one or two conservative amino acid substitutions (only V↔I or R↔K)! The TriPaxB penetratin RVVQVWFQNQRAKLKK from the *Trichoplax adhaerens* protein Lim1 (UniProt entry B5LDT8) served as a query (named TriPaxB) for extended penetratins in other simple organisms (see Table 2).

*T. adhaerens* has a high regeneration and rejuvenation potential, partially due to the regulated expression of homeotic genes Not and Trox-2 [92]). The best-conserved regions of corresponding proteins contain penetratin-like peptides A**Q**V**K**V**WFQNRR**I**KW**R**K** and K**Q**V**KIWFQNRR**V**KWKK**.We used the bold font for residues from the *T. adhaerens* peptides are identical to Drosophila pAntp penetratin residues.

Example 4: Poriferans

The Pax-6 protein XP_003387530.1 (or Uniprot entry A0A1X7UM72) from the embryo of the sponge *Amphimedon queenslandica* is annotated as the homeobox domain-containing protein (by UniProt) and as paired box protein Pax-6-like (by NCBI genome annotation data). In both databases, the DNA binding is predicted as the transcription factor activity. The PaxB penetratin from *T. adhaerens* with the sequence ARVQVWFSNRRAKWRK is similar to the SRVQVWFQNRRAKWRK peptide in the sponge’s Pax-6. Substituted residues are in bold font and underlined.

Example 5: Amoeboid protist

The amoeboid holozoan *Capsaspora owczarzaki* is one close unicellular relative of animals [84]. Authors labeled as Co_5 the homeobox domain from the protein A0A0D2VSA1. It contains six arginines within the penetratin sequence RVIRIWFQNRRAKQRR. Other natural penetratins have a high number of Arg and Lys residues (Table 1). These sequences are still underexplored candidates for transporting bioactive cargo into the cell.

### 3.2. The Penetratin-like Cryptides from Other Kingdoms

The search among ascomycetes (fungi) also resulted in diverse penetratins. One hit with the Pax-6 annotation is for the *Ceratocystis platani* fungus causing disease on sycamore trees. It is the Paired box protein Pax-6 (KKF93291.1) with 639 residues. The penetratin analog from its homeobox region has a 56% identity to pAntp penetratin (see Table 1).

Another regulatory protein PHO2 (A0A1E5RMZ3) with the homeobox domain from Hanseniaspora osmophila (wine-making yeast) has an associated penetratin analog, which is similar in its sequence TQVKIWFQNRRMKWKR to the pAntp. The budding yeast penetratin analog KNVRIWFQNRRAKVRKKGKL extended at its C-terminal (underlined) from the PHO2 (Q6FKZ3) protein has a high positive charge and unknown abilities. Its CPP probability prediction by the MLCPP server is similar (0.93) to pAntp (0.98). Hemolytic activity prediction by the HAPPENN server is a strikingly low probability of 0.018 compared to pAntp’s 0.936. Thus, exploring natural penetratin analogs from all available sources can be the first stepping stone toward discovering nontoxic CPP candidates with a peptide backbone.

Two representative bacterial and one archeon species are included in Table 1 because at least one homeobox domain-containing motif with penetratin analog is found among their expressed proteins. The similarity is modest or low to pAntp. Archeon penetratin analog **RQ**VSV**WF**T**N**A**R**KRIWL is only 38% identical to pAntp penetratin (residues with bold font are 6 out of 16 residues), raising doubts about similar functions.

Some viral proteins contain remarkably efficient CPP, such as the TAT peptide from HIV [93,94], which has as promising drug-delivery therapeutic potential as penetratin [95]. The TAT peptide sequence GRKKRRQRRRPPQ is, however, easily cleaved by furin. Thus, CPP is not stable enough in vivo for efficient cargo delivery [96]. Hemmati et al. [97] identified 310 decapeptides with predicted CPP activity in the proteome of severe acute respiratory syndrome coronavirus 2 (SARS-CoV-2). In the surface glycoprotein S (spike protein) alone, there are 24 CPP candidates, some rich in Arg residues. Nucleocapsid protein N is even richer in CPP candidates (54). Arginines are required firstly for binding to negatively charged groups of viral nucleic acid [98] and secondly for penetrating the eukaryotic cell membrane.

**Figure 1:**
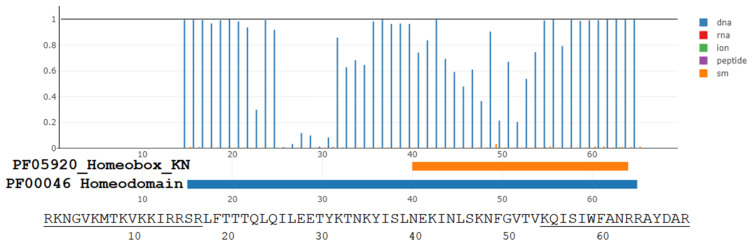
The dSPRINT server [47] prediction for DNA-binding probabilities (vertical axis, blue lines profile) of residues from a homeodomain found in a giant virus *Moumouvirus maliensis* protein QGR53678.1. Probabilities are negligible for binding residues to RNA, ions, other peptides, and small molecules (other colors for profile lines). See the main text for details on the Pfam domains PF05920 and PF00046. We added the query sequence below the graph produced by the dSPRINT server. The underlined residues are the predicted CPP segment (N-terminal) and the penetratin-like peptide (C-terminal).

The superkingdom of viruses includes the class of giant viruses. The genomes with accession numbers: NC_014649, NC_020104, and NC_016072 contain homeobox proteins. The dSPRINT server [47] examines whether the protein domain query binds DNA, RNA, small molecules, ions, or peptides and assigns corresponding interaction probabilities to each interaction type for each residue. Figure 1 illustrates these probabilities for predicted CPP peptide and penetratin analog present within the homeodomain-containing protein QGR53678.1 of a giant *Moumouvirus maliensis* virus. The corresponding residues Arg-44 to Arg-112 with underlined Table 1 peptides for that virus are: RKNGVKMTKV(10)KKIR**RSR****LFT**(20)**T**TQ**L**QILEET(30)YKTNK**YISLN**(40)EK**I**NLSKNFG(50)VTVK**QI**S**IWF**(60)**ANRRA**YDAR,where we highlighted with a bold font those residues for which DNA-binding probability is higher than 0.95. The probability of binding ligands other than DNA is less than 0.05 for all residues within both predicted homeodomain motifs. Thus, three C-terminal residues from the predicted CPP peptide (underlined N-terminal 17 residues) and ten residues from the predicted penetratin analog (underlined C-terminal 16 residues) are strongly predicted DNA-binding residues (Figure 1).

There are many predicted CPP cryptides from giant viruses other than penetratin analogs. For example, the MLCPP and C2Pred servers predict with a high probability (0.94 and 0.96) that the RKNGVKMTKVKKIRRSR sequence (see Figure 1) should have the CPP activity. We can adopt a tentative name 9RK17 for that CPP cryptide, which is hidden in a putative homeodomain from the GenBank entry QGR53678.1 at a different sequence location from the penetratin analog KQISIWFANRRAY*D*ARK. We doubt that all CPP cryptides from giant viruses (such as 9RK17) have been examined in experiments for their cargo-transporting efficiency inside eukaryotic cells. For instance, the 21 amino acid long cryptide ALHARRRRARQRLCQHRVSIK is present in the hypothetical *Pandoravirus dulcis* (giant virus) protein YP_008318537.1. The predicted CPP probability is 0.95 (MLCPP server) and 0.90 (C2Pred server). A longer cryptide MTWRRSCWRLLRQRRRQPRSPKMMRKR is the N-terminal of hypothetical peptide YP_001425938.1 encoded by the *Paramecium bursaria Chlorella virus* FR483 genome (also a giant virus). The peptide has associated CPP probability predictions of 0.94 and 0.99 by MLCPP and C2Pred server.

Some bacteria and viruses tolerate the differences in the last four residues of natural penetratin analogs (such as W14 to D14 substitution). These residues are less critical for interaction with DNA. Examples of W14 to D14 substitution in penetratin-like peptides from the homeobox domain are found in human sequences, too (see Homeobox even-skipped homolog proteins 1 and 2 with the UniProt links P49640 and Q03828).

The penetratin’s biological role in a homeodomain is to serve as a major aggregation site for DNA-binding residues. The same is likely to hold for all other presented Table 1 sequences. The dSPRINT server finds the same GO: 0003677 molecular function by which a gene product interacts selectively and non-covalently with DNA for these sequences. For corresponding proteins, the dSPRINT server finds PF00046_Homeodomain, PF05920_Homeobox_KN motif, or both motifs overlapping the penetratin analog. One example is the N-terminal part with 60 residues of the *Euryarchaeota archaeon* RYE98021.1 protein. For residues 11–40, the prediction for the PF05920_Homeobox_KN motif is associated with the E-value of 3.2 × 10^−10^. For residues 25–54, the prediction with the E-value of 1.8 × 10^−8^ is for the PF00046_Homeodomain motif. The hexadecapeptide sequence RQVSVWFTNARKRIWL extends from Arg-18 to Leu-33, thus forming a part of both homeobox motifs. Extended sequence RQVSVWFTNARKRIWLPLRQKQARMRNKRAK, with residues 18–48, has a higher CPP probability score of 0.93. Therefore, CPP, DNA-binding ability, and the transcription factor DNA-binding function are frequently present in the same protein domains.

The UniProt database of all known and predicted proteins contains 85,650 sequences from 1394 species with the PF00046_Homeodomain annotation. While Table 1 is far from comprehensive, it still reports several additional species from Megaviricetes compared to the Brandes and Linial data analysis in 2019 [99]. It is, of course, due to the fast progress in genetic sequencing. An astonishing universality of that Pfam family motif in Animalia, Fungi, Protista, Eubacteria, Archaea, and Viruses indicates its conservation across almost all of life’s superkingdoms and kingdoms.

The PF05920_Homeobox-KN Pfam domain (Figure 1, thick orange line below the x-axis) is also universal in all kingdoms of life. It belongs to the conserved homeobox transcription factor KN domain from TALE, KNOX, and MEIS genes [100]. Current Pfam taxonomy does not mention the presence of the PF05920_Homeobox-KN motif in bacteria and viruses.

A caveat to keep in mind for penetratin-like peptides from bacteria, archaea, and viruses is the hypothetical or predicted nature of some proteins containing them. Low annotation scores in public databases may lead to failed verification for claimed associated species.

### 3.3. The Translocation Function of Homeobox Proteins, Homeobox, Penetratin, and Penetratin-like Peptides

Homeodomain proteins fulfill many biological functions for which other segments in these proteins are also crucial. The unconventional transport mechanism for these proteins is an active research area [101]. Direct translocation of an identical protein in and out from eukaryotic cells is complex because eukaryotic plasma membranes are asymmetric. Their internal lipid layer has a different lipid composition from the external layer. Neutral polar lipids, such as phosphatidylcholine, prevail among phospholipids oriented (with their head groups) toward the cell exterior. Negatively charged phospholipids, such as phosphatidylserine, are plentiful only among polar lipids in contact with the cell cytoplasm. Moreover, fatty acids’ unsaturation in the cytoplasmic plasma membrane leaflet is about twofold higher [102]. In the case of engrailed-2 homeoprotein transfer, the anionic phospholipid phosphatidylinositol-4,5-biphosphate is also involved [103]. It is a minor component of the plasma membrane inner leaflet [104] and even less frequent in the outer leaflet. Still, it is essential as a gatekeeper for cell signaling and molecular traffic among cells [105]. Moreover, cell surface carbohydrates are probably involved in the cellular uptake of homeoproteins from the external environment [106]. Therefore, the ability of such proteins for unconventional bidirectional transfer across the plasma membrane of some eukaryotic cells is likely to rely on distinct mechanisms for outside-directed and inside-directed transport.

Distinct mechanisms imply the existence of several dedicated protein motifs for targeting the plasma membrane from the cytoplasm and the cell outside. Specifically, the bidirectional transfer function must be in-built inside an extended penetratin-like region for each homeodomain segment. Dupont et al. [107] examined whether the penetratin extended in its N-terminal to encompass the turn region between the second and third helix is enough to ensure the peptide transport in and out of cells. Dupont et al. [107] named it the SecPen peptide QSLAQELGLNE**RQIKI****WFQNRRMKWKK**, where the Sec peptide is underlined, and the penetratin domain is highlighted with bold font.

The QSLAQELGLNE Sec peptide is a cryptide in engrailed-2 proteins Q05917 (HME2_CHICK), P52730 (HME2B_XENLA), and P09015 (HME2A_DANRE), to mention only the reviewed Swiss-Prot proteins containing that peptide. The human analog of the QSLAQELGLNE peptide contains glycine to serine substitution. Sec and Pen allow for bidirectional membrane crossing [106]. These and other authors verified the validity of the signaling homeoproteins concept with far-reaching implications [108].

Homeoproteins are rich in multifunctional cryptides. For example, let us examine the UNIPROT Q05917 entry and structurally solved PDB 3ZOB sequence 3ZOB_1 with three α-helices [109] for chicken engrailed 2 homeoprotein. The GAG (glycosaminoglycans at the cell surface)-binding sequence **P**(186)**RSRKPKKKNPN**KEDKRPR(204) is located just before chicken engrailed 2 homeodomain (residues 200–259). That highly flexible protein region contains two CW BBXB quadruplets (Cardin-Weintraub motifs [110]) and one KKK triplet, all described as glycosaminoglycan or heparan sulfate binding motifs [111]. The bold font for the residues at the N-terminal highlight the motif, which is part of the putative nuclear localization signal (see Figure 1B from reference [111]). It is also a DNA-binding motif, which has a significant probability of penetrating cells (0.88, according to the MLCPP server). Thus, the multiplicity of functions for crucial motifs from engrailed proteins is more a rule than an exception.

Among other examples, the N-terminal hexapeptide QRRSRT for the Pax3 and Pax7 homeodomain is also a good starting point for the design of multifunctional peptides. We can ask what would be predicted activities for the sequence tandem peptide QRRSRTGQRRSRT with inserted Gly residue as a middle flexible linker. That tridecapeptide is expected to be nontoxic by the Raghava ToxinPred server [38], highly cell-penetrating (the MLCPP server), and strongly DNA-binding (binding probability higher than 0.7 for all arginines according to the DP-BIND server [36]). However, predictions by the CAMP_R3_ and AmpGram algorithms exclude its antimicrobial function. When we fuse the QRRSRTGQRRSRT sequence with some antimicrobial peptide such as IKKIVSKIKKLLK (L-K6V1-temporin-1CEb) [112], it can gain multifunctional abilities without undesirable hemolytic and toxic effects. For instance, the hybrid peptide with the sequence KKLFKKILKYL-GG-QRRSRTGQRRSRT (BP100-CPP conjugate) is expected to have all six considered functions and lesser hemolytic activity compared to BP100. The same idea should work for N-terminal decapeptide GLNRRRKKRT from the homeobox domain of the pou2f1 transcription factor (Xenopus laevis African clawed frog, Uniprot entry P16143). The sequence tandem GLNRRRKKRTGLNRRRKKRT did not need middle Gly insertion, its cell-penetrating probability score of 0.98 is almost maximal, and all residues 3 to 19 of that 20 residues long peptide have DNA-binding probability higher than 0.8. Moreover, the tandem peptide may have antimicrobial activity against intracellular pathogens. The CAMP_R3_ server SVM module result is 0.925 probability for the AMP activity, while the HAPPEN server predicts a negligible probability of 0.03 for the hemolytic activity.

The translocation function is the best researched for the homeobox protein engrailed-2 from chicken, which is 99% identical to human En2 [109,111]. However, for chick and human engrailed-2 protein, the hexadecapeptide analog of Drosophila antennapedia penetratin is different in underlined residues: SQIKIWFQNKRAKIKK (only one arginine instead of three). A decreased number of arginines opens the question about the importance of human and chick penetratin motifs for membrane translocation of corresponding homeodomain and intact engrailed proteins.

The previous paragraphs indicated that the translocation function might be mediated by protein motifs outside the homeobox domain acting in concert with the recognition helix from that domain. Suppose a minimal number of six consecutive arginines is needed for cell penetration [113]. In that case, the question is whether these residues are close in the 3D structure but not so close in sequence. Hence, we can speculate that CPP activity can be preserved after the number of arginines drops to the single one within the penetratin-like peptides during biological evolution with a compensatory increase in strategically placed arginines outside penetratin.

Firstly, it is easy to find cases when more arginines are in the homeodomain regions preceding the penetratin segment. Secondly, space separation may exist among negative and positive charges. Anionic residues (D and E) may be located only at the one homeodomain surface. The residues with positive charges dominate at the opposite homeodomain surface where the penetratin motif is situated. The spatial separation of anionic from cationic charges persists for the engrailed 2 protein when one examines only two last homeodomain helices with a turn between them. Thus, an electrostatic dipole moment and the corresponding electric field are more substantial for the whole homeodomain and the 2nd-helix-turn-3rd-helix compared to penetratin peptides, which are mostly devoid of negative charges.

We have recently published the observation that strong 3D electrostatic and 3D-hydrophobic moments are instrumental for better interaction between some flexible cationic peptides with helix-turn-helix secondary structures and membranes containing polar lipids with anionic head groups [114]. The calculated hydrophobic moment for an ideal α-helix rod (the 2D moment) is not relevant for the peptide–membrane interaction of highly plastic peptides such as penetratin [115]. Furthermore, a high degree of peptide helicity or amphipathicity is not required for penetratin internalization [116].

The helix-turn-helix motif of engrailed proteins is the ultrafast independently folding domain [117]. An additional internalization advantage for intact homeodomain is that its 20 times lower extracellular concentration of 5 × 10^−8^ M is enough to achieve substantial accumulation in the cell nuclei [118]. In contrast, micromolar penetratin concentrations must be added for efficient internalization [49].

Three arginines from the pAntp penetratin RQIKIWFQNRRMKWKK are not the only regulators of its translocation process. The substitution of two tryptophans with similarly bulky aromatic and hydrophobic phenylalanine residues inhibits penetration internalization [119]. The role of two tryptophans has been examined in the tryptophan fluorescence study after the first (Trp-6) or second Trp (Trp-14) has been substituted with the Phe residue [49]. The first Trp from the wild-type penetratin sequence motif WF inserts more deeply into the lipid bilayer than the second Trp. The WF motif is also better conserved across biological kingdoms (Table 1). Penetratin membrane incorporation is more profound in the presence of anionic polar lipids, such as phosphatidylserine.

To study the cell penetration mechanism, direct interaction with specific plasma membrane phospholipids is as essential for penetratin-like peptides as their binding to glycosaminoglycans at the cell surface. The mechanism and target molecules may differ among penetratin analogs, homeoboxes, and homeoproteins. We previously mentioned the involvement of phosphatidylinositol-4,5-biphosphate [103], a key lipid signaling molecule important for endocytosis, exocytosis, membrane fusion, and myriad other biological activities. In addition to cell-surface GAGs and heparan sulfate, polysialic acid is also the surface receptor for pAntp Drosophila homeobox peptide [118].

Lysines are less critical for penetratin uptake compared to arginines. When all lysines are replaced with arginines, a designed analog sequence RQIRIWFQNRRMRWRR-NH_2_ exhibits almost 50% better internalization ability than wild-type penetratin [55]. Wild-type penetratin possesses moderate antimicrobial activity [50]. In comparison, Bahnsen et al. [55] found that the analog with seven arginines has about four times stronger antimicrobial activity against *E. coli*. However, the analog exhibits eight times greater toxicity to human cells. These activity changes are not predicted by the servers we used (compare results for pAntp peptide 1 from Table 2 and PenArg peptide 1 from Table 3). On the other hand, predictions and experimental validations agree that amphipathic antimicrobial peptides with high lysine content can have negligible hemolytic activity and low toxicity. One example is L-K6V1-Temporin-1CEb [112] (Table 3, peptide 40).

Electrostatic interactions are important for translocation into cells [120]. These interactions have been tuned during biological evolution by clustering positive charges near the C-terminal of penetratin-like peptides and by retaining lone arginine at the first or second N-terminal position in animals. The lengthwise charge asymmetry is accompanied by the hydrophobic interactions of peptide middle leading to the bend conformation parallel to the membrane surface.

Detailed molecular dynamics simulations and free energy calculations uncovered the role of Trp-6 interaction with Arg-1 and Arg-10 at the membrane surface [121]. In observed Trp-Arg stacking, the indol ring of W is positioned almost parallel to the guanidinium group of R. Trp-6 is more involved than Trp-14—the observation of the importance of WR cation–π interactions [122], which is in accordance with the better preservation of Trp−6 in penetratin-like peptides. We can safely assume that all of the presented penetratin-like sequences from Table 1 (and many more not present in that table) are membrane-active peptides. The membrane-activity terminology implies that peptide conformational plasticity and membrane curvature adaptation occurs after mostly disordered peptides from an aqueous solution reach the membrane surface [53,121,123,124]. The structural plasticity of penetratin (from random coil to beta-sheet and α-helix in different environments) is relatively high among other cell-penetrating peptides [125]. It contributes to its functional CPP versatility through clathrin-mediated endocytosis, caveolae-mediated endocytosis, macropinocytosis, and direct translocation by forming inverted micelles [53,126,127].

Clathrin-mediated endocytosis is an active transport process requiring GTP hydrolysis [128]. On the other hand, direct translocation is an energy-independent uptake. It is a self-initiated spontaneous process producing only transient perturbation of plasma membrane integrity [116]. Alves et al. [53] proclaimed: “penetratin usurps endocytotic cell processes but can also translocate into the cells.” Translocation and uptake rates depend on CPP sequence and concentration, cell type, buffer, temperature, cargo (if any), and other experimental variables [56]. With such versatility, it is no wonder that penetratin can induce phase separation, de-packing of membrane lipids, negative curvature, and aggregation of lipid vesicles [123,129]. These macroscopic effects of penetratin are enhanced for cases of higher membrane fluidity and the presence of anionic phospholipids at the membrane surface.

One biological role of penetratin is the contribution to driving the translocation of its parent homeoprotein, but the translocation of intact homeoprotein is much more efficient (<1 nM [106]) in comparison with the penetratin uptake. Homeoproteins are natural cargoes for at least some penetratin-like peptides. Moreover, homeoproteins are active cargoes with non-penetratin protein regions participating in the synergetic amplification of specific translocations. The biological roles have not been examined for most of the natural penetratin-like peptides. That did not prevent widespread penetratin usage in life sciences and therapeutic applications.

### 3.4. Penetratin Sequence Optimization and Possible Applications

Penetratin sequence optimization by Kauffman et al. [56] resulted in considerably improved direct translocation (with different cargoes) by the RKKRWFRRRRPKWKK analog with six arginines, five lysines, and two tryptophans. Similarly designed penetratin analogs may be helpful delivery vehicles for biotechnological applications and systemic therapeutics (a fast-growing market). Older results on the vectorization strategies with penetratin are gathered in the book by Dupont et al. [130].

The mechanisms of CPP penetration and CPP-cargo transport across the blood–brain barrier are discussed this year by Zorko and Langel [131]. Penetratin is usually linked with a drug, protein, or nucleic acid cargo at its N-terminal. Škrlj et al. [132] used penetratin as the linker peptide connecting two antibody fragments specific for the pathological form of the prion protein. That vectorization strategy enabled efficient delivery across the blood–brain barrier. Liposomal formulation using penetratin molecules is an effective treatment strategy for delivering a therapeutic gene to the brain. The aim is, for instance, to reverse Alzheimer’s disease pathophysiology [133]. Non-viral gene delivery for all therapeutic goals has advantages when penetratin or similar peptides are used as nontoxic vehicles that do not provoke an immune response.

In the proof of principle experiments, Liu et al. [134] demonstrated how penetratin-coated nanoparticles can reach the eye fundus, thus eliminating the need for invasive eye injection during the gene therapy treatment of diseases such as diabetic retinopathy and age-related macular degeneration. Needle-in-the-eye application is naturally associated with low patient compliance and increased infection risk.

The penetratin (PEN) and other cell-penetrating peptides have a promising potential for drug targeting and oncological pharmacotherapy [57,58]. Combating drug-resistant cancers by targeted delivery of drugs should facilitate the development of effective personalized therapies. The designed GEM-PEN conjugate improved the intracellular delivery and anticancer activity of gemcitabine (GEM) [135]. Anticancer peptides can also be covalently connected to penetratin. Kanovsky et al. [136] synthesized three p53 peptides PPLSQETFS, PPLSQETFSDLWKLL, and ETFSDLWKLL in peptide linkage to reversed penetratin analog sequence KKWKMRRNQFWVKVQRG. The authors did not explain their rationale for reversing the Antennapedia penetratin sequence G**RQIKIWFQNRRMKWKK** (in the bold font) or replacing isoleucines with valines with added terminal glycine. It is connected to the previous observation about the absence of chiral receptor requirement for the transduction ability of penetratin and its reversed analog (see the publication [137] cited by Kanovsky et al. [136]). The three p53 peptides are amino-terminal parts of that tumor suppressor protein, which can interact with oncogene-encoded ubiquitin-protein ligase mdm-2 (MDM2 [Q00987]), targeting p53 for degradation and accelerated proliferation of cancer cells.

Kanovsky et al. [136] reasoned that the blockage of p53-mdm-2 interactions could inhibit cell-transforming oncogenic events by competition of the peptides mentioned above to p53 for mdm-2 binding. Thus, these three peptides should be able to act as anticancer if they can reach intracellular mdm-2 target proteins. The attachment of reverse penetratin KKWKMRRNQFWVKVQRG sequence to the carboxy-terminal end of each peptide had a dual role—to enable transport of the peptides across the plasma membrane and to stabilize the α-helical conformation of each peptide for maximal interaction with mdm-2 proteins. NMR experiments subsequently confirmed the helical conformation [138] (see the PDB entry 1Q2F). Increased helical content of the peptide was not achieved when the penetratin leader sequence was attached to the amino-terminal end of the PPLSQETFSDLWKLL sequence. It resulted in considerably lower helical probabilities of reverse penetratin carboxy-terminal part (with added Gly residue) and bioactive peptide amino-terminal segment containing the Pro pair. Therefore, the N-terminal or C-terminal conjugation of a bioactive peptide to CPP is not arbitrary. It should be guided by the maximization of the interaction with internal targets of chimeric peptides. Chosen peptide conjugates by Kanovsky et al. [136] were highly cytotoxic on various tumor cells and did not affect normal cells in culture.

Interestingly, amino-terminal p53 peptides induce cell death in malignant cells without inducing apoptosis and independently of p53 protein activation, arguing for a general antiproliferative effect on these cells. The software tools ACPred and mACPred failed to predict the high probability of anticancer function for reverse VV–penetratin hybrid with N-terminal p53 peptide PPLSQETFS (see Table 2, peptide 11). Hence, the p53 peptide conjugated to penetratin was erroneously classified as noncancer (NACP).

Selivanova et al. [139] examined the option for C-terminal p53 peptides conjugated to penetratin. The importance of the p53 gene stems from observations that more than half of human tumors have mutations in that gene. Transcribed protein has several DNA binding domains. The G(361)SRAHSSHLKSKKGQSTSRHKK(382) sequence is the most highly charged cationic domain near the C-terminal (see P04637 UniProt entry), which regulates DNA binding. Selivanova et al. [139] investigated whether the C-terminal peptide can restore the growth suppressor function of mutant p53 proteins. The authors used the peptide **GSRAHSSHLKSKKGQSTSRHKK**WKMRRNQFWVKVQRG (named fusion peptide 46; see peptide 19 predictions in Table 2). By bold font and underlining, we highlighted the C-terminal p53 peptide and reversed penetratin to emphasize that CPP is ligated to the carboxy-terminal end of the bioactive peptide without its KK pair at the amino-terminal end because the KK pair is already present at the C-terminal of the fusion peptide.

Subekti and Kamagata [140] proposed the role of the flexible and disordered C-terminal p53 domain. It enables p53 to land on and twin around DNA, forming the encounter complex at lower salt concentrations. The flexibility facilitated the protein jumping along DNA at higher salt concentrations. Selivanova et al. [139] proved that the growth suppressor function of mutant p53 could be restored by an excess of the fusion peptide 46. The authors proposed that the peptide can displace the C-terminal domain from its binding site to the core p53 domain.

Restoring the ability to bind DNA worked for Ala-143, His-175, Trp-248, Ser-249, His-273, and Lys-280 mutant forms of p53 [141]. Activated p53 induced apoptosis in Ew36 and BL41 Burkitt lymphoma cells, SW480 colon carcinoma cells, and breast cancer cells MCF-7, MDA-MB-468, and MDA-MB-231, despite mutant p53 forms being present in these cells [141]. Normal breast and colon cell lines were not affected. The corresponding peptide 19 from Table 2 has predicted DNA-binding, cell-penetrating, antimicrobial, antiviral, and antifungal activity combined with toxicity absence by some of the algorithms we used. However, peptide 19 is associated with modest probabilities of 0.61 and 0.65 for anticancer activity as calculated by the ACPred [26] and mACPpred [27] servers. Of course, experimental results should prevail in our minds over any theoretical predictions. We can anticipate the therapeutic benefits of anticancer-peptide-CPP conjugates when their pharmacokinetic parameters are improved for medical applications.

### 3.5. Multifunctional or Hybrid Penetratin-like Peptides

Table 2 results belong to three peptide classes. The first class contains natural sequences 1 (pAntp), and 3 (TriPaxB). Listed examples of longer natural peptides 4–6 with additional four residues at each peptide terminal contain the TriPaxB penetratin and belong to the second class. The first sequence (peptide 4 in Table 2) is from an uncharacterized cnidarian protein with 445 AA from medusa Clytia hemisphaerica (jellyfish). The following peptide (peptide 5) is found in the T2M9B9 UniProt entry for an unreviewed protein named LIM homeobox transcription factor 1-alpha (LMX1A). The protein LMX1A is from the fresh-water polyp Hydra vulgaris, claimed to be immortal [142,143]. The sequence for peptide 6 (A0A183IGD8) is from the parasitic stomach-dwelling worm of American martens Soboliphyme baturini and Loa loa eye worm. These three natural sequences were submitted to the dSPRINT server http://protdomain.princeton.edu/dsprint (accessed on 7 August 2022) [47]. They have a common PF00046_Homeodomain motif for the first 20 residues and the GO: 0003677 molecular functions by which a gene product interacts selectively and non-covalently with DNA. Rationally designed peptides 2 and 7–22 are the third class. Peptide 2 is the VV-penetratin sequence RQVKVWFQNRRMKWKK. It is present in the predicted homeobox proteins of some birds and fishes (UniProt entries A0A7K7IKL9, A0A7K9GUV0, and A0A1A8LZ63). The designed sequences validated in experiments have the “/E” extension in their abbreviated name. In silico design by this author is associated with the “/DJ” extension.

Regarding possible penetratin involvement in antimicrobial defense, Drosophila pAntp penetratin RQIKIWFQNRRMKWKK-NH_2_ is fungicidal for the clinical isolates of *Cryptococcus neoformans* [51]. It exhibits moderate antibacterial activity against *Escherichia coli* and *Staphylococcus aureus* with MIC values from 32 to 64 μM [55]. Some of penetratin’s natural analogs from Table 1 may have stronger antimicrobial potency or better therapeutic index. Our goal was to find or design multifunctional peptides with low predicted toxicity to healthy human cells. All Table 2 peptides have predicted cell-penetrating and DNA-binding activity combined with a considerably lower prediction for the hemolytic activity compared to pAntp penetratin. In addition, most Table 2 peptides have predicted antimicrobial, anticancer, antiviral, antifungal, and anti-inflammatory activity. For sequences 4–6, 11, and 14–15, the ACPred server does not predict anticancer activity. Some of them have been designed and validated as ACP (peptide 11).

It is not easy to achieve strongly predicted antifungal (probability higher than 0.7) along with other activities and low toxicity to red blood cells. At the end of Chapter 2, we explain our reasons for choosing the higher limit of 0.83 for hemolytic activity probability, which can still ensure good selectivity. The peptides 2–11, 13–18, and 20–22 from Table 2 satisfy that criterion. Three of them are constructs involving parts of the pexiganan antibiotic and TriPaxB or VV-penetratin (peptides 7–9). Peptide 10 is fused TriPaxB with the antifungal sequence BP16 studied by Badosa et al. [144]. Peptide 13 is reversed VV-penetratin [136] fused to the anticancer TPR peptide [145]. The Gly residue is a flexible linker between two bioactive peptides in both cases. The N-terminal part of peptide 15 is reversed amoebae penetratin (peptide 14 from Table 2), which we singled out in Table 1 as a natural penetratin-like peptide with the highest number of arginines (six). Short C-terminal sequence CGIKRTK is similar to tumor-homing peptide tLyp-1 with the sequence CGNKRTR [146]. The tLyp-1 and CGIKRTK are nontoxic but also not associated with other predicted activities except cell penetration (see peptide 1 from Table 5).

The optimization for better anti-inflammatory activity led to the best multifunctional peptides 20 (with underlined activity scores) and 21 from Table 2. They consist of a reverse penetratin analog [56] (see peptides 16 and 17) with two amino acid substitutions (A8 and I15) and analogs to the tumor-homing peptide [146]. The predicted toxicity to red blood cells is very low (0.01) for peptides 20 and 21. Another advantage of these peptides is their short length (22 residues). Their overall rank among all 176 sequences from Table 2, Table 3, Table 4 and Table 5 is 6th and 22nd. Peptide 21 is an example of when increasing the number of substitutions to increase the anti-inflammatory activity impairs other functionalities. The peptide 22 is an analog of reversed optimized penetratin [56] (see Chapter 4 for details of its design). Its overall rank is 31st (Table 6). Still, its short length (18 residues) and predicted lack of hemolytic activity and toxicity argue for experimental validation of cell-penetrating, antibacterial, anticancer, and antiviral activity.

The tentative conclusions from Table 2 are the following. Searching through natural cryptides from biological databases is always a promising initial approach. Using the rational design may be more successful in widening the activity spectrum of bioactive-CPP conjugates. In vitro and in vivo tests can confirm whether some of Table 2 peptides remain viable candidates for drug development. For a hybrid pAntp–TPR anticancer sequence (peptide 12), predicted hemolytic activity slightly decreases in comparison with pAntp alone. The observed toxicity of peptide 12 to normal cell lines is significantly smaller than its toxicity to cancer cell lines [145].

If confirmed, the antifungal activity might be the most interesting for several reasons. Firstly, nature’s design for penetratins gives these peptides the specialized ability to easily pass through the eukaryotic cell membrane and for DNA binding. Secondly, there are precious few drugs toxic to fungal cells causing different diseases but are nontoxic to human cells. One example is the urgent need for compounds inhibiting the growth of *C. neoformans* yeasts in patients who had organ transplantation and are immunocompromised. Thirdly, the conjugated antifungal–CPP hybrid peptide may gain additional activities, as predicted in Table 2 (see peptide 10). The rational design option for creating antifungal hybrid peptides targeting intracellular molecules is to conjugate penetratin or some penetratin analog with known antifungal peptides such as LKLFKKILKVL or KKLFKKILKKL [144]. They are active against pathogenic fungi Fusarium oxysporum. The probability for antifungal activity increased from 0.22 for the TriPaxB penetratin sequence RVVQVWFQNQRAKLKK (see Table 2, peptide 3) to 0.54 or higher for the constructs RVVQVWFQNQRAKLKK-G-LKLFKKILKVL or RVVQVWFQNQRAKLKK-G-KKLFKKILKKL (see Table 2, peptide 10 for the second construct predictions). The sequence should be submitted to other predictive algorithms (besides iAMPpred [31] and AntiFungal [32]) for serious consideration of experimental confirmations.

Confusingly, a dedicated server for the classification of peptides according to predicted antifungal activity—the http://webs.iiitd.edu.in/raghava/antifp (accessed on 7 August 2022) server, predicts as non-antifungal the peptides LKLFKKILKVL (BP33; [144]), KKLFKKILKKL (BP16; [144]), LKLFKKILKVLG, together with hybrid peptides LKLFKKILKVL-G-RVVQVWFQNQRAKLKK, RVVQVWFQNQRAKLKK-G-LKLFKKILKVL, and sequence 10 from Table 2.

**Table 2:** Hybrid penetratin-like peptides with predicted DNA binding, CPP, antimicrobial, anticancer, antiviral, antifungal, anti-inflammatory, hemolytic, and toxic activity.

No.	Peptide/Gene/Origin *	Extended TriPaxB or Reverse Penetratin/Sequence Number *	DNA- Bind. **	CPP ^$^	Anti- Microbial ^‡^	Anti- Cancer ^$$^	Anti- Viral ^&^	Anti- Fungal ^⁋^	Anti-Inflamm. Activity ^§^	Hemo- lytic ^¥^	Toxicity/Score ^†^
1	P02833/*D. melanogaster* penetratin/E	**RQIKIWFQNRRMKWKK** /339–254/pAntp	+	0.998/H	0.97/0.42	0.812/0.985	1.0/0.70/0.77	0.28/0.95	0.57/0.68	0.94/+	−0.66
2	Rev. VV-pen. [136]/E	**KKWKMRRNQFWVKVQR**	+	0.956/H	0.96/0.53	0.649/0.981	0.10/0.44/0.28	0.15/0.68	0.52/0.66	0.19/−	−0.81
3	TriPaxB penetratin	**RVVQVWFQNQRAKLKK**	+	0.807/L	0.74/0.42	0.036/0.971	0.00/0.40/0.01	0.22/0.21	0.52/0.61	0.02/−	−1.42
4	A0A7M5V8Y3/N/A */Clytia hemisphaerica*	GLSV**RVVQVWFQNQRAKLKK**IQKK/227–250	+	0.642/L	0.96/0.32	0.189/0.980	0.44/0.47/0.82	0.56/0.59	0.65/0.62	0.03/−	−1.45
5	T2M9B9/UP ^&&^/*Hydra vulgaris*	GLSV**RVVQVWFQNQRAKLKK**LHRK/227–250 and 108–131	+	0.761/L	0.93/0.37	0.065/0.983	0.66/0.46/0.97	0.58/0.45	0.66/0.61	0.03/−	−1.16
6	A0A1S0TPC1/UP ^&&^/ *Loa loa*	NLSV**RVVQVWFQNQRAKLKK**IQRK/118–141	+	0.715/L	0.91/0.29	0.049/0.769	0.21/0.57/1.0	0.28/0.59	0.67/0.63	0.04/−	−1.47
7	PexNC-TriPaxB-I/DJ	GIGK-**RVVQVWFQNQRAKLKK**-ILKK	+	0.731/L	0.99/0.67	0.968/0.980	0.93/0.75/1.0	0.97/0.59	0.61/0.58	0.09/−	−1.51
8	PexShort-TriPaxB-II (PexT)/DJ	GIGKLKKAKKFGKKILKK-G- **RVVQVWFQNQRAKLKK**	+	0.792/L	1.0/0.98	0.995/0.951	0.98/0.76/0.52	1.0/0.61	0.64/0.58	0.13/−	−1.17
9	PexNC-rev. VV-pen./DJ	GIGK-G-**KKWKMRRNQFWVKVQR**-ILKK	+	0.849/H	1.0/0.58	0.919/0.982	1.0/0.65/0.95	0.94/0.93	0.55/0.67	0.26/−	−1.17
10	TriPaxB–antifungal BP16 [144]/DJ	**RVVQVWFQNQRAKLKK-G**- KKLFKKILKKL	+	0.816/L	0.98/0.95	0.992/0.981	1.0/0.70/0.93	0.98/0.54	0.54/0.64	0.62/+	−1.46
11	Anti-cancer-I-Rev. VV-pen. [136]/E	PPLSQETFS- **KKWKMRRNQFWVKVQRG**	+	0.503/H	0.41/0.53	NACP/NACP	0.40/0.90/1.0	0.15/0.05	0.62/0.62	0.13/−	−1.09
12	pAntp-TPR [145]/E	**RQIKIWFQNRRMKWKK-** KAYARIGNSYFK	+	0.834/H	0.91/0.50	0.923/0.939	1.0/0.80/0.54	0.83/0.65	0.59/0.62	0.91/+	−1.09
13	Rev.-VV-pen. [136]-TPR/DJ	**KKWKMRRNQFWVKVQR**-G- KAYARIGNSYFK	+	0.766/H	0.83/0.48	0.766/0.952	1.0/0.79/1.0	0.87/0.40	0.60/0.63	0.59/+	−1.13
14	Rev. amoeba *(Filasterea*) pen. with added N-term-Arg/DJ	**RRQKARRNQFWIRIVRR**	+	0.958/H	1.0/0.46	0.110/0.984	0.01/0.27/0.73	0.44/0.71	0.59/0.62	0.07/−	−0.58
15	Rev. R-am.pen.-tLyP-1 [146]/DJ	**RRQKARRNQFWIRIVRR**- **CGIKRTK**	+	0.962/H	0.98/0.51	0.259/0.984	0.78/0.91/0.93	0.88/0.43	0.68/0.62	0.02/−	−0.88
16	Optimal penetratin (o-pen P14 [56]/E	**RKKRWFRRRRPKWKK**	+	0.992/H	1.0/1.0	0.767/0.978	0.97/0.57/1.0	0.32/1.0	0.47/0.56	0.02/−	−0.89
17	Rev. opt. penetratin (r-o-p)/DJ	**KKWKPRRRRFWRKKR**	+	0.992/H	1.0/0.99	0.767/0.980	1.0/0.92/1.0	0.32/1.0	0.48/0.49	0.01/−	−1.11
18	Rev.opt.pen. (r-o-p)-tLyP-1 [146]/DJ	**KKWKPRRRRFWRKKR**- **CGIKRTK**	+	0.987/H	0.94/1.0	0.854/0.979	1.0/0.82/0.59	0.71/0.96	0.65/0.68	0.006/−	−1.30
19	Fusion peptide 46 [139]/E	GSRAHSSHLKSKKGQSTSRH- **KKWKMRRNQFWVKVQRG**	+	0.741/L	0.76/0.87	0.61/0.65	0.98/0.48/0.15	0.81/0.44	0.67/0.59	ND	−0.89
**20**	**Rev.opt.pen. (r-o-p A_8_I_15_)-tLyP-1** [146]**-analog1/DJ**	**KKWKPRRARFWRKKI**- **CGIKRTK**	+	0.987/H	0.96/0.993	0.972/0.98	1.0/0.8183/0.77	0.89/0.922	0.6674/0.647/ 1.397	0.007/−	−1.39
21	Rev.opt.pen. A_8_I_15—_ tLyPA_3_-1-analog2/DJ	**KKWKPRRARFWRKKI**- **CGAKRTK**	+	0.985/H	0.97/0.99	0.943/0.981	1.0/0.78/0.27	0.86/0.96	0.66/0.66/ 1.56083	0.006/−	−1.22
22	Rev. optimized penetratin analog/DJ	**GKRIGKKWKPRRRRFWRK**	+	0.991/H	1.0/1.0	0.944/0.979	1.0/0.96/0.95	0.59/1.0	0.61/0.61	0.003/−	−1.26

* Highlighted peptides (bold name) with underlined activity scores are our selection for the designed peptides with the best overall score (see Table 6). All peptides are assumed to be amidated at their C-terminal. Letter ‘E’ after peptide name means that the sequence has been synthesized and tested in experiments. DJ abbreviation means that according to our knowledge, we were the first to find or design that peptide. Bold sequence segments have predicted or verified CPP activity. Underlined residues are optimal substitutions for increasing anti-inflammatory activity or decreasing peptide toxicity. ** The results of DP-Bind server http://lcg.rit.albany.edu/dp-bind/ (accessed on 7 August 2022) by Hwang et al. [36] for sequence-based prediction of DNA-binding residues in DNA-binding proteins. The “+” sign means that the server found several DNA-binding residues. ^$^ The probability that the peptide is cell-penetrating peptide (CPP) or non-CPP (NCPP) with the MLCPP server http://www.thegleelab.org/MLCPP/ [22]. Predicted high and low uptake efficiency is denoted with, respectively, letters ‘H’ and ‘L’. ^‡^ Antimicrobial peptide probabilities with CAMP_R3_ Support Vector Machine algorithm of the server http://www.camp.bicnirrh.res.in/predict (accessed on 7 August 2022) [24] and with AmpGram (http://biongram.biotech.uni.wroc.pl/AmpGram/ [25]. ^$$^ The ACPred server (http://codes.bio/acpred/ [26] is used to classify peptides as anticancer (ACP) or non-anticancer (NACP) with a given probability. The mACPred server (http://thegleelab.org/mACPpred/ [27] results for the probability of anticancer activity are added after the ‘/’ symbol. ^&^ Results of peptide antiviral prediction with servers ENNAVIA (https://research.timmons.eu/ennavia [28], sequence length restricted between 7 and 40 residues)/FIRM-AVP (https://msc-viz.emsl.pnnl.gov/AVPR/ (accessed on 7 August 2022) [29]/Meta-iAVP (http://codes.bio/meta-iavp/ (accessed on 7 August 2022) [30]. ^⁋^ Results of iAMPpred peptide antifungal prediction by Meher et al. [31] (http://cabgrid.res.in:8080/amppred/server.php, (accessed on 7 August 2022)) and Zhang et al. [32] (https://www.chemoinfolab.com/antifungal/, (accessed on 7 August 2022)). ^§^ Results for the prediction of anti-inflammatory activity (Anti-inf.) by the AIPpred (first number; http://www.thegleelab.org/AIPpred/ (accessed on 7 August 2022) [33], PreAIP (second number; http://kurata14.bio.kyutech.ac.jp/PreAIP/ (accessed on) [34] server, and the score output of the AntiInflam server (http://metagenomics.iiserb.ac.in/antiinflam/ (accessed on 7 August 2022) [35] server when it predicts the anti-inflammatory activity. ^¥^ The probability that the peptide has hemolytic activity by the HAPPENN server [40] https://research.timmons.eu/happenn (accessed on 7 ugust 2022). After the peptide name, we introduced the |lcl|cTer term to obtain the prediction for the amidated C-terminal. Symbols ‘+’ and ‘−’ are used for peptide classification as hemolytic or not. ^†^ Toxicity prediction by the ToxinPred server https://webs.iiitd.edu.in/raghava/toxinpred/ (accessed on 7 August 2022) [37,38,39]. We used batch submission for peptides [37]. The design module of that server was used when we wished to optimize the peptide for decreased toxicity after several amino acid substitutions. ^&&^ UP = Uncharacterized protein.

**Table 3:** CPP bioactive peptide conjugates for intracellular targets I. Activity probabilities.

No.	Peptide or Parent Protein/ Gene/Origin/Reference *	CPP Constructs/Sequence Number *	CPP	Anti-Microbial	Anti-Cancer	Anti-Viral	Anti-Fungal	Anti-Inflamm.	Hemo- lytic	Toxicity/ Score
1	PenArg (Bahnsen-2013 [55])/E	**RQIRIWFQNRRMRWRR**	0.99/H	0.99/0.57	0.32/0.98	1.0/0.7/0.4	0.24/0.96	0.60/0.66	0.94	−1.12
2	DiR_6_WF OLQ14316.1/ *S. microadriaticum*	**RRRRRRWFRRRRRRWFRKI** /603–621 DiR_6_WF	0.99/H	1.00/0.97	0.92/0.91	1.0/0.3/1.0	0.43/0.82	0.57/0.59	0.68	−0.97
3	WFR_8_ from CellPPD ^$^ scan of DiR_6_WF/DJ	**RRWFRRRRRR**	0.99/H	1.00/0.99	0.95/0.98	0.9/0.6/0.9	0.42/ND	0.53/0.53	0.21	−0.92
4	Reverse WFR_8_ (R_8_FW)/DJ	**RRRRRRFWRR**	0.99/H	1.00/0.89	0.95/0.98	0.9/0.4/0.9	0.42/ND	0.56/0.53	0.08	−0.93
5	Ribos.-hom.-pept. (RHP)-pAntp/ [54]/E	YKWYYRGAA- **RQIKIWFQNRRMKWKK**	0.90/H	0.74/0.46	0.95/0.98	1.0/0.9/0.8	0.49/0.68	0.64/0.62	0.97	−0.63
6	HK2-WFR_8_ [147]/DJ	MIASHLLAYFFTELN-GG- **RRWFRRRRRR**	0.80/H	0.62/0.19	0.15/0.99	1.0/0.8/1.0	0.17/0.45	0.62/0.59	0.30	−1.28
7	RHP [54] -WFR_8_/DJ	YKWYYRGAA-**RRWFRRRRRR**	0.97/H	1.0/0.97	0.85/0.98	1.0/0.8/1.0	0.63/0.93	0.60/0.63	0.12	−1.10
8	RtLyp-1-G-VV-pen. ^&^/DJ	**RCGNKRTR**-G- RQVKVWFQNRRMKWKK	0.94/H	0.78/0.49	0.12/0.98	1.0/0.5/1.0	0.57/0.83	0.58/0.61	0.24	−0.67
**9**	**L-K6V1 temporin 1CEb** [112]-**GG-WFR_8_/DJ**	IKKIVSKIKKLLK-GG-**RRWFRRRRRR**	0.97/H	0.98/1.00	0.97/0.98	1.0/1.0/1.0	0.97/1.00	0.54/0.67/1.0796	0.17	−1.35
10	CAMEL [148]-WFR_8_/DJ	KWKLFKKIGAVLKVL- **RRWFRRRRRR**	0.96/H	1.00/1.00	0.81/0.98	1.0/1.0/1.0	0.82/1.00	0.61/0.66	0.98	−1.33
11	Rev. WFR_8_–CAMEL [148]/DJ	**RRRRRRFWRR**-GG- KWKLFKKIGAVLKVL	0.96/H	1.00/1.00	0.73/0.98	1.0/0.9/1.0	0.92/0.98	0.60/0.63	0.71	−1.33
12	[R4, R10]-chensinin-1b [149]- WFR_8_/DJ	VWRRWRRFWRR-GG- **RRWFRRRRRR**	0.99/H	1.00/0.95	0.93/0.98	1.0/0.7/0.1	0.41/0.99	0.58/0.71	0.72	−1.02
13	ZY4 [150]-GG-WFR_8_/DJ	VCKRWKKWKRKWKKWCV-GG- **RRWFRRRRRR**	0.98/H	0.99/1.00	0.91/0.98	1.0/0.9/0.8	0.40/1.00	0.53/0.68	0.60	−0.50
14	Puroindoline [151]-WFR_8_/DJ	FPVTWRWWKWWKG-G- **RRWFRRRRRR**	0.99/H	1.00/1.00	0.87/0.98	1.0/0.9/1.0	0.21/0.99	0.61/0.66	0.85	−0.99
15	Rev. WFR_8—_puroindoline/DJ	**RRRRRRFWRR**-GG- FPVTWRWWKWWKG	0.98/H	1.00/0.95	0.85/0.98	1.0/0.9/1.0	0.23/0.99	0.61/0.62	0.49	−1.01
16	Novispirin [152] -WFR_8_/DJ	KNLRIIRKGIHIIKKY-GG- **RRWFRRRRRR**	0.95/H	1.00/1.00	0.95/0.98	1.0/1.0/1.0	0.94/0.99	0.63/0.62	0.53	−1.14
17	BP33 antifungal [144]/E	LKLFKKILKVL	0.85/H	0.84/1.00	1.0/0.98	1.0/0.5/1.0	0.98/1.00	0.48/0.65	0.57	−1.30
18	BP33 antif. [144]-pAntp/DJ	LKLFKKILKVL-G- **RQIKIWFQNRRMKWKK**	0.86/H	1.00/0.92	0.98/0.98	1.0/1.0/1.0	0.98/1.00	0.54/0.66	1.0	−1.09
19	TriPaxB-antifungal-BP33 [144]-with-GGG-tag/DJ	**RVVQVWFQNQRAKLKK**- LKLFKKILKVL-GGG	0.62/H	0.96/0.95	0.84/0.84	0.9/0.9/1.0	0.98/0.23	0.64/0.65	0.63	−1.58
20	rWFR_8_-antif-BP16 [144]/DJ	**RRRRRRFWRR**-GG- KKLFKKILKKL	0.982/H	1.00/1.00	0.97/0.98	1.0/0.7/1.0	0.90/1.00	0.57/0.68	0.57	−1.40
21	T2R1 [88]-WFR_8_/DJ	RHHWRRYARIGFRAVRTVIGK-G- **RRWFRRRRRR**	0.901/H	1.00/1.00	0.73/0.97	1.0/1.0/0.9	0.70/0.90	0.71/0.64	0.30	−1.18
22	WFR_8_-DiPGLa-H [153]/DJ	**RRRRRRFWRR**-G- KIAKVALKALKIAKVALKAL	0.970/H	1.00/1.00	0.57/0.97	1.0/0.9/1.0	0.91/0.99	0.64/0.66	0.80	−1.09
23	WFR_8_-TPR [145] with G_4_ link/DJ	**RRWFRRRRRR**-GGGG- KAYARIGNSYFK	0.893/H	1.00/0.73	0.71/0.98	1.0/0.6/1.0	0.90/0.76	0.59/0.57	0.16	−1.38
24	GV1001 vaccine [154]-WFR_8_/DJ	EARPALLTSRLRFIPK-GG- **RRWFRRRRRR**	0.951/H	1.00/0.59	0.95/0.98	1.0/1.0/1.0	0.66/0.95	0.69/0.74	0.05	−1.35
25	BP100 [155]-WFR_8_/DJ	KKLFKKILKYL-GG- **RRWFRRRRRR**	0.981/H	1.00/1.00	0.45/0.98	1.0/1.0/1.0	0.92/0.97	0.60/0.69	0.71	−1.39
26	RWBP100 [156]-WFR_8_/DJ	RRLFRRILRWL-GG- **RRWFRRRRRR**	0.994/H	1.00/0.97	0.84/0.98	1.0/0.8/1.0	0.53/0.99	0.61/0.70	0.65	−1.23
27	Mitochondrial targeting [157]- WFR_8_/DJ	KLLNLISKLF-GGG-**RRWFRRRRRR**	0.938/L	1.00/0.98	0.43/0.98	1.0/0.9/0.8	0.81/0.99	0.62/0.67	0.82	−1.31
28	Nosangiotide [158]-WFR_8_/DJ	RKKTFKEVANAVKISA-GG- **RRWFRRRRRR**	0.917/H	0.98/0.96	0.25/0.97	0.9/0.9/0.9	0.79/0.95	0.67/0.58	0.08	−1.09
29	Buforin [159] -WFR_8_/DJ	TRSSRAGLQFPVGRVHRLLRK- GGG-**RRWFRRRRRR**	0.945/H	0.99/0.87	0.05/0.98	1.0/0.9/0.4	0.91/0.99	0.68/0.60	0.04	−0.89
30	Buforin-BR2 [160]/E	RAGLQFPVGRLLRRLLR	0.879/L	1.00/0.71	0.42/0.98	0.8/0.9/1.0	0.20/1.00	0.53/0.63	0.01	−1.12
31	BR2-WFR_8_/DJ	RAGLQFPVGRLLRRLLR-GG- **RRWFRRRRRR**	0.960/H	1.00/0.97	0.43/0.98	1.0/0.9/1.0	0.68/1.00	0.53/0.63	0.25	−1.23
32	WFR_8_-Zp3a [161]/DJ	**RRWFRRRRRR**-GIKAKIGIKIKK	0.98/H	0.99/1.00	0.84/0.98	1.0/0.8/1.0	0.89/0.98	0.53/0.66	0.07	−1.25
33	RHP [54]-rev. WFR_8_/DJ	YKWYYRGAA-**RRRRRRFWRR**	0.97/H	1.00/0.80	0.85/0.98	1.0/0.9/1.0	0.63/0.95	0.61/0.62	0.04	−1.03
34	T2R3G3/DJ	RRRHHWRRYARIGFRAVRTVIGK-GGG	0.87/H	0.99/0.84	0.85/0.97	1.0/0.9/1.0	0.85/0.54	0.67/0.66	0.06	−1.19
**35**	**Temporin-asparagutin analog1/DJ**	IKKIVSKILKLLKV-G-**RRWFRRRRRR**	0.96/H	0.998/1.00	0.96/0.98	1.0/1.0/1.0	0.96/1.0	0.60/0.71/1.625	0.76	−1.47
**36**	**Temporin-asparagutin analog2/DJ**	IKKIVSKIRKLLK-GG-**RRWFRSRRRR**	0.96/H	0.92/0.99	0.96/0.98	1.0/1.0/1.0	0.97/0.99	0.62/0.66/1.5	0.18	−1.30
**37**	**Temporin-asparagutin analog3/DJ**	VKKIVSKIRKLLK-GG-**RRWFRSRRRR**	0.97/H	0.92/0.99	0.95/0.98	1.0/1.0/1.0	0.96/0.99	0.63/0.64/1.72	0.13	−1.27
38	Novispirin [152]-WFR_8_-analog1/DJ	KNLRLIRKGIHIILKY-GG-**RRWFLRRRRR**	0.938/H	1.0/1.0	0.768/ 0.981	1.0/0.9854/1.0	0.96/0.995	0.5814/0.648/1.5622	0.551	−1.17
39	Temporin-1CEb [162]/E	ILPILSLIGGLLGK	0.453	0.789/1.0	0.991/ 0.984	0.101/0.083/0.68	0.98/0.97	0.47/0.62	0.959	−1.08
40	L-K6V1-Temporin-1CEb [112]/E	IKKIVSKIKKLLK	0.880/L	0.930/1.0	1.0/0.982	0.991/0.589/0.40	0.87/1.0	0.53/0.64	0.009	−1.20
41	T2R1 [88]/E	RHHWRRYARIGFRAVRTVIGK	0.907/H	0.973/0.617	0.96/0.984	0.999/0.901/0.89	0.67/0.764	0.63/0.63	0.017	−1.06
42	Rev. WFR_8_-hinge-aurein 1.2 [3]/DJ	**RRRRRRFWRR**-GGGPPK- GLFDIIKKIAESF	0.817/H	0.941/0.994	0.897/0.916	1.0/0.874/1.0	0.94/0.988	0.609/0.575	0.082	−1.02
43	SVS-1 [163]/E	KVKVKVKVDPLPTKVKVKVK	0.817/L	0.96/0.746	0.962/0.973	0.0/0.4/0.344	0.48/0.978	0.43/0.41	0.003	−0.84
44	HPRP-A1-TAT [6,164]/E	FKKLKLFSKLWNW-K**RKKRQRRR**	0.975/H	0.997/0.997	0.527/0.984	1.0/0.928/0.972	0.69/0.987	0.586/0.669	0.043	−0.98
45	Beclin-1-R11 [165]/E	TNVFNATFEIWHDGEFGT- **RRRRRRRRRRR**	0.814/H	0.987/0.034	0.516/0.846	0.83/0.834/0.36	0.26/0.268	0.565/0.552	0.005	−0.92
46	Mapegin [88]/E	KIGKKILKALKGALKELA	0.707/H	0.588/1.0	1.0/0.982	0.783/0.589/0.988	0.98/1.0	0.59/0.67/1.55745	0.079	−1.32
47	MAP [166]/E	**KLALKLALKALKAALKLA**	0.998/H	0.794/1.0	0.979/0.986	0.345/0.096/0.918	0.42/1.0	0.54/0.73/0.69540	0.973	−1.13
48	Mapegin-TAT/DJ	KIGKKILKALKGALKELA- **GRKKRRQRRRPPQ**	0.878/H	0.929/0.997	0.764/0.981	0.998/0.975/0.506	0.96/1.0	0.65/0.65/1.52487	0.026	−1.04
49	Mapegin-a1-TAT/DJ	KIGKKILKALKLALKLLA- **GRKKRRQRRRPPQ**	0.958/H	0.980/1.0	0.613/0.983	0.998/0.975/0.630	0.94/1.0	0.67/0.76/2.13612	0.717	−1.09
**50**	**Mapegin-a2-TAT/DJ**	KITKKILKALKGALKELA- **GRKKRRQRRRMPQ**	0.881/L	0.518/0.994	0.717/0.942	0.998/0.93/0.972	0.97/0.996	0.68/0.65/1.53687	0.077	−1.81

* We used the servers listed in Table 2 and applied them in the same order for columns CPP to Toxicity. Highlighted peptides (bold name) with underlined activity scores are our selection for the designed peptides with the best overall score (see Table 6). Bold sequence segments have predicted or verified CPP activity. Underlined residues are optimal substitutions for increasing anti-inflammatory activity or decreasing peptide toxicity. ^$^ The best CPP candidates from longer peptides were found by using the protein scanning CellPPD (http://crdd.osdd.net/raghava/cellppd/ (accessed on 7 August 2022) [167]. ^&^ See peptides 1 and 2 from Table 5 for the origin, references, and abbreviations of cancer-homing tLyP-1 peptides and their analogs.

**Table 4:** CPP bioactive peptide conjugates for intracellular targets II. Activity probabilities *.

No.	Peptide Name/Ref.	Extended CPP at the N or C-terminal *	CPP	Anti-Microbial	Anti-Cancer	Anti-Viral	Anti-Fungal	Anti-Inflamm.	Hemo-lytic	Toxicity/ Score
1	KW [168]/E	**KRKRWHW**	0.99/H	1.00/ND	0.98/0.98	0.8/0.4/1.0	0.38/ND	0.62/0.54	0.01	−0.93
2	Ribosomal-homing- peptide (RHP)-KW [54]/DJ	YKWYYRGAA-**KRKRWHW**	0.93/H	0.97/1.00	0.99/0.98	1.0/0.9/0.8	0.62/0.74	0.51/0.61	0.02	−0.58
3	L-K6V1 temp [112]-KW/DJ	IKKIVSKIKKLLK-GG-**KRKRWHW**	0.89/H	0.98/1.00	1.0/0.98	1.0/1.0/0.2	0.95/1.00	0.59/0.66	0.02	−1.35
4	CAMEL [148]-KW/DJ	KWKLFKKIGAVLKVL-**KRKRWHW**	0.92/H	1.00/1.00	0.99/0.98	1.0/1.0/1.0	0.78/0.99	0.61/0.67	0.94	−0.90
5	R_2_-chensenin [149]-KW/DJ	VWRRWRRFWRR-GG-**KRKRWHW**	0.99/H	1.00/0.99	0.95/0.98	1.0/0.9/1.0	0.27/1.00	0.66/0.70	0.15	−1.03
6	ZY4 [150]-KW/DJ	VCKRWKKWKRKWKKWCV-GG- **KRKRWHW**	0.95/H	1.00/1.00	0.99/0.98	1.0/0.9/1.0	0.34/1.00	0.58/0.68	0.23	−0.36
7	Puroindoline [151]-KW/DJ	FPVTWRWWKWWKG-G-**KRKRWHW**	0.88/H	1.00/0.99	0.98/0.98	1.0/0.9/1.0	0.46/0.99	0.64/0.65	0.67	−0.83
8	Novispirin [152]-KW/DJ	KNLRIIRKGIHIIKKY-GG-**KRKRWHW**	0.90/L	0.98/1.00	0.99/0.98	1.0/1.0/1.0	0.96/1.00	0.63/0.63	0.43	−0.90
9	BP33 [144]-KW/DJ	LKLFKKILKVL-G-**KRKRWHW**	0.93/H	1.00/1.00	0.99/0.98	1.0/1.0/1.0	0.92/1.00	0.62/0.70	0.82	−1.19
10	T2R1 [88]-KW/DJ	RHHWRRYARIGFRAVRTVIGK- **KRKRWHW**	0.94/H	0.99/0.94	0.92/0.98	1.0/1.0/0.8	0.71/0.86	0.66/0.62	0.12	−1.09
11	DiPGLa-H [153]-KW peptide/DJ	KIAKVALKALKIAKVALKAL- **KRKRWHW**	0.92/L	0.98/1.00	0.99/0.98	1.0/1.0/1.0	0.97/0.99	0.49/0.63	0.94	−0.92
12	Neoepitope4-WFR_8_ [169]/DJ	VLSHGSFVM-GG-**RRWFRRRRRR**	0.89/H	0.89/0.93	0.59/0.98	1.0/0.8/0.1	0.62/0.76	0.62/0.62	0.43	−1.22
13	WFR_8_ -tumor homing [170]/DJ	**RRWFRRRRRR**-GG-IFLLWQR	0.99/H	1.00/0.78	0.48/0.98	1.0/0.5/0.8	0.46/0.96	0.63/0.63	0.07	−1.26
14	BP100 [155]-KW/DJ	KKLFKKILKYL-GG-**KRKRWHW**	0.93/H	1.00/1.00	1.0/0.98	1.0/1.0/1.0	0.94/0.99	0.58/0.65	0.61	−1.35
15	Mitoch. target. [157]-KW/DJ	KLLNLISKLF-GGG-**KRKRWHW**	0.80/L	0.97/1.00	0.74/0.98	1.0/1.0/0.9	0.91/0.98	0.63/0.67	0.41	−1.29
16	Nosangiotide [158]-KW/DJ	RKKTFKEVANAVKISA-GG-**KRKRWHW**	0.69/L	0.85/0.93	0.87/0.97	0.5/0.9/0.9	0.88/0.84	0.69/0.59	0.01	−1.03
17	Adepantin-1A [88]-WFR_8_/DJ	GIKKAVGKALKGLKGLLKALGES -GG-**RRWFRRRRRR**	0.80/L	1.00/0.99	0.95/0.98	1.0/1.0/1.0	0.98/1.00	0.60/0.66/1.30566	0.66	−1.46
**18**	**WFR_8_-adepantin-1A/DJ**	**RRWFRRRRRR**- GIKKAVGKALKGLKGLLKALGES	0.86/L	1.00/1.00	0.95/0.96	1.0/1.0/1.0	0.97/0.99	0.62/0.62/1.36028	0.63	−1.56
19	KW-pexiganan-L18 [88]/DJ	**KRKRWHW**- GIGKFLKKAKKFGKAFVLILKK	0.87/H	0.99/1.00	1.0/0.98	1.0/0.9/1.0	0.99/0.99	0.53/0.64	0.81	−1.04
20	RtLyp-1-flexampin [114]/DJ	R**CGNKRTR**- GIKKWVKGVAKGVAKDLAKKIL	0.59/L	0.92/1.00	1.0/0.97	1.0/1.0/1.0	1.00/1.00	0.44/0.63	0.68	−0.74
21	Zyk-1- [88]-WFR_8_/DJ	GIGREIIKKIIKKIGKKIGRII -GG-**RRWFRRRRRR**	0.89/H	1.00/1.00	0.99/0.98	1.0/1.0/1.0	0.96/0.99	0.60/0.66	0.88	−1.18
22	MG2-bombesin [171]/E	GIGKFLHSAKKFGKAFVGEIMNS-GG- QRLGNQWAVGHLM	0.30	0.83/0.85	0.86/0.54	1.0/0.9/0.9	0.97/0.41	0.53/0.55	ND	−0.97
23	MG2-pAntp [172]/E	GIGKFLHSAKKFGKAFVGEIMNS-GG- **KKWKMRRNQFWVKVQRG**	0.52/L	0.95/1.00	0.93/0.81	1.0/1.0/1.0	0.99/0.81	0.56/0.52	ND	−0.68
24	DP1 [173]/E	RRQRRTSKLMKR-GG- KLAKLAKKLAKLAK	0.95/L	0.84/1.00	0.91/0.98	0.7/0.7/0.2	0.95/0.55	0.50/0.65	0.03	−0.36
**25**	**KW-BMAP-18** [174]**/DJ**	**KRKRWHW**-GGLRSLGRKILRAWKKYG	0.90/H	1.00/1.00	0.98/0.98	1.0/1.0/1.0	0.88/0.98	0.58/0.68/1.3653	0.09	−1.01
26	Chrysophin-1-KW [175]/DJ	FFGWLIKGAIHAGKAIHGLI-GG- **KRKRWHW**	0.59/L	0.99/1.00	0.98/0.98	1.0/1.0/1.0	0.96/0.97	0.52/0.55	0.97	−1.03
27	KW-mastoparan [176]/DJ	**KRKRWHW**-GG-INLKALAALAKKIL	0.90/L	0.87/1.00	0.75/0.98	1.0/0.9/1.0	0.92/0.96	0.62/0.66	0.42	−1.11
28	KW-pleuricidin [177]/DJ	**KRKRWHW**- GWGSFFKKAAHVGKHVGKAALTHYL	0.66/L	0.89/1.00	0.99/0.98	1.0/0.9/0.9	0.96/1.00	0.60/0.65	0.37	−0.95
29	MTD [178]/E	**RRRRRRRRGRQ**-KLLNLISKLF	0.98/H	0.28/0.60	0.58/0.96	1.0/0.6/0.9	0.73/0.97	0.67/0.70	0.06	−1.06
**30**	**L-K6V1 temp** [112]**-KW-analog/DJ**	IKKIVSKI**R**KLLK**R**-G-**KRKRWHW**	0.95/H	0.98/1.00	1.0/0.98	1.0/1.0/0.8	0.92/1.0	0.65/0.66/1.678	0.07	−1.12
31	T2R1 [88]-KW-analog1/DJ	RHHWRRYARIGFRAVRSVIGK- **KTKRWHW**	0.92/H	0.93/0.94	0.98/0.98	1.0/1.0/1.0	0.69/0.97	0.66/0.62/1.36406	0.03	−1.09
**32**	T2R1 [88]-KW-analog2/DJ	RHHWRRLARIGFRAVRSVIGK- **KTKRWHW**	0.93/H	0.96/0.87	0.97/0.98	1.0/1.0/1.0	0.60/0.97	0.67/0.62/1.5722	0.05	−1.30
**33**	**KW-BMAP-18** [174]**-analog1/DJ**	**KRKRWHW**-GGLRSLGRKLLRAWKKYG	0.91/H	1.00/0.99	0.96/0.98	1.0/1.0/1.0	0.84/0.97	0.63/0.71/1.62236	0.08	−1.05
34	BP100 [155]-KW-analog/DJ	**L**KLFKKILKYL**N**-G-**KRKRWHW**	0.93/H	0.996/0.999	0.966/0.981	1.0/0.961/1.0	0.87/1.0	0.635/0.687/1.80571	0.894	−1.32
35	Zyk-1 [88]-WFR_8_-analog/DJ	GIG**L**EI**V**KKII**L**KIGKKIGRII-GG- **RRWFRRRRRR**	0.83/L	0.999/0.998	0.986/0.977	1.0/0.985/0.964	0.98/0.99	0.60/0.614/1.599	0.938	−1.33
**36**	**KW-BMAP-18** [174]**-analog2/DJ**	**KRKRWHW**-GGL**A**SLGRK**L**LRAWKKYG	0.85/H	0.988/0.986	0.951/0.982	1.0/0.971/1.0	0.85/0.97	0.684/0.708/1.85648	0.399	−1.09
**37**	**R_8_FW-GGGPPKG-** **temp** [112] **R_9_R_14_/DJ**	**RRRRRRFWRR**-GGGPPKG- IKKIVSKI**R**KLLK**R**	0.95/H	0.997/1.0	0.954/0.968	1.0/0.958/0.822	0.97/0.99	0.71/0.60/1.25133	0.032	−1.18
**38**	**R_8_FW-GGEPPKG-** **temp** [112] **R_9_R_14_/DJ**	**RRRRRRFWRR**-GGEPPKG- IKKIVSKI**R**KLLK**R**	0.94/H	0.998/0.988	0.926/0.973	1.0/0.96/0.996	0.96/0.944	0.70/0.596/1.51442	0.028	−1.20
**39**	**R_7_A_5_FW-GGEPPKG temp** [112]**/DJ**	**RRRRARFWRR**-GG**E**PPKG- IKKIVSKIRKLLKR	0.92/H	0.998/0.968	0.916/0.973	1.0/0.9654/0.95	0.97/0.927	0.72/0.597/1.80807	0.010	−1.25
40	L-K6V1 temp. [112]-GGEPPKG-KW/DJ	IKKIVSKIKKLLK-GGEPPKG- **KRKRWHW**	0.72/L	0.971/0.986	0.996/0.955	0.994/0.985/0.97	0.94/0.839	0.50/0.624	0.007	−1.15
41	R_8_FW-GGGPPKG-IDR-1002 [9]/DJ	**RRRRRRFWRR**-GGGPPKG- VQRWLIVWRIRK	0.97/H	1.0/0.944	0.181/0.979	1.0/0.862/0.854	0.71/0.40	0.633/0.606	0.064	−1.17
42	R_8_FW-GGGPPKG-IDR-1018 [9]/DJ	**RRRRRRFWRR**-GGGPPKG- VRLIVAVRIWRR	0.95/H	1.0/0.978	0.344/0.980	1.0/0.863/0.982	0.79/0.33	0.612/0.602	0.039	−1.17
43	R_8_FW-GGGPPKG- IDR-1018-R_6_/DJ	**RRRRRRFWRR**-GGGPPKG- VRLIV**R**VRIWRR	0.96/H	1.0/0.929	0.406/0.980	1.0/0.832/0.354	0.81/0.59	0.626/0.605	0.027	−1.21
44	R_8_FW-GGEPPKG-IDR-1018-R_6_/DJ	**RRRRRRFWRR**-GG**E**PPKG- VRLIV**R**VRIWRR	0.96/H	1.0/0.702	0.356/0.983	1.0/0.854/0.99	0.78/0.21	0.612/0.607/1.16498	0.026	−1.23
45	R_8_FW-GGEPPKG-IDR-1018-L_1_R_6_/DJ	**RRRRRRFWRR**-GG**E**PPKG- **L**RLIV**R**VRIWRR	0.96/H	1.0/0.939	0.277/0.983	1.0/0.86/0.04	0.77/0.338	0.623/0.622/1.43256	0.025	−1.20
46	Pexiganan-L18 [88]/E	GIGKFLKKAKKFGKAFVLILKK	0.75/L	0.997/1.0	1.0/0.976	0.46/0.299/0.22	1.0/1.0	0.598/0.661	0.892	−0.94
47	Flexampin [114]/E	GIKKWVKGVAKGVAKDLAKKIL	0.56/L	0.990/1.0	1.0/0.977	0.993/0.937/0.544	0.99/1.0	0.423/0.531	0.817	−0.78
48	Zyk-1 [88]/E	GIGREIIKKIIKKIGKKIGRII	0.65/L	0.978/0.998	0.998/0.97	1.0/0.933/0.946	0.88/1.0	0.526/0.662	0.583	−0.86
49	Adepantin-1A [88]/E	GIKKAVGKALKGLKGLLKALGES	0.39	0.980/1.0	1.0/0.977	1.0/0.972/0.398	0.99/1.0	0.554/0.659/1.35587	0.17	−1.51
**50**	**Novispirin** [152]**-KW-analog2/DJ**	KNLRI**F**RKGIHI**H**KKY-GG- **KRKRWHW**	0.903/H	0.972/0.946	0.994/0.983	1.0/0.939/0.822	0.96/0.989	0.5884/0.6	0.195	−1.63
**51**	**WFR_8_-adepantin-1A-** **analog2/DJ**	**RRWFRRRRRR**- GIKKAVGKALKGLK**L**LLKALGES	0.878/L	0.999/1.0	0.923/0.9485	1.0/0.987/0.908	0.96/0.983	0.616/0.622/1.62411	0.826	−1.63
52	KW-second-bovine- BMAP-18 [179]/DJ	**KRKRWHW**-GRFKRFRKKFKKLFKKIS	0.961/H	0.999/1.0	0.995/0.981	1.0/0.845/1.0	0.91/1.0	0.565/0.698	0.176	−1.09

* We used the servers listed in Table 2 and applied them in the same order. Highlighted peptides (bold name) with underlined activity scores are our selection for the designed peptides with the best overall score (see Table 6). Bold sequence segments have predicted or verified CPP activity. Underlined residues are optimal substitutions for increasing anti-inflammatory activity or decreasing peptide toxicity.

**Table 5:** Activity probabilities for CPP conjugated magainin analogs, MF constructs, and Arg-Pro rich peptides *.

No.	Parent-Protein/Gene/ Origin/Reference *	Extended CPP at the N or C-terminal *	CPP	Anti-Microbial	Anti-Cancer	Anti-Viral	Anti-Fungal	Anti-Inflamm.	Hemo -lytic	Tox./Score
1	Tumor-homing-tLyP-1 peptide [146]/E	**CGNKRTR**	0.91/L	0.00/ND	N/0.88	N/N/N	ND/ND	0.38/0.47	0.01	−0.42
2	A7RG57 C-term. from *N. vectensis*	**RCGIKRTK**	0.93/L	0.03/ND	0.93/0.95	0.6/0.5/0.5	0.92/ND	0.47/0.62	0.00	−0.88
3	MFC/DJ	RCGNKRFRWHW	0.94/H	0.43/0.91	0.97/0.98	1.0/0.8/1.0	0.38/0.98	0.47/0.63	0.01	−0.92
4	NLS-CE [180]/E	WRFVWMNPKKKRKV	0.92/H	0.99/0.54	0.46/0.98	0.8/0.5/0.8	0.13/0.76	0.47/0.59	0.11	−1.11
5	Zp3a [161]/E	GIKAKIGIKIKK	0.77/L	0.94/1.00	1.0/0.97	0.2/0.1/0.3	0.86/1.00	0.48/0.63	0.03	−0.68
6	Magainin 2 (MG2) [181]/E	GIGKFLHSAKKFGKAFVGEIMNS	0.22	0.95/1.00	1.0/0.98	1.0/1.0/1.0	0.99/0.98	0.56/0.55	0.83	−0.58
7	MG2-tLyP-1 [146]/DJ	GIGKFLHSAKKFGKAFVGEIMNS-GG- **CGNKRTR**	0.26	0.88/0.99	0.99/0.93	1.0/1.0/1.0	0.99/0.94	0.53/0.52	0.76	−0.35
8	MG2-KW [168]/DJ	GIGKFLHSAKKFGKAFVGEIMNS-GG- **KRKRWHW**	0.43	0.94/1.00	0.99/0.96	1.0/1.0/1.0	0.99/0.92	0.57/0.52	0.63	−0.68
9	MG2-WFR_8_/DJ	GIGKFLHSAKKFGKAFVGEIMNS-GG- **RRWFRRRRRR**	0.74/L	0.93/1.00	0.95/0.97	1.0/1.0/1.0	0.98/0.99	0.61/0.52	0.72	−0.90
10	9P0-1 [182]/E	GIKKWLHSAKKFGKKFVKKIMNS	0.72/L	0.99/1.00	1.0/0.98	0.8/0.9/1.0	0.99/0.98	0.61/0.64	0.96	−0.42
11	MFC-9P0-1-analog [182]/DJ	**RCGNKRFRWHW**- GIKKWLHSAKKFGKKFVKKIMNS	0.76/H	0.92/1.00	1.0/0.93	1.0/1.0/0.9	0.95/0.96	0.63/0.70	0.86	−0.59
12	MFC-Zp3a [161]/DJ	**RCGNKRFRWHW**-GIKAKIGIKIKK	0.89/H	0.98/0.99	0.98/0.98	1.0/0.7/0.9	0.97/1.00	0.57/0.68	0.01	−1.05
13	9P1-3 [182]/E	GIKKWLHSAKKFPKKFVKKIMNS	0.73/L	0.99/1.00	1.0/0.98	0.9/0.9/0.6	0.97/0.98	0.63/0.64	0.94	−0.30
14	MFC-9P1-3 [182]/DJ	**RCGNKRFRWHW**- GIKKWLHSAKKFPKKFVKKIMNS	0.78/H	0.88/1.00	1.0/0.92	1.0/1.0/0.8	0.93/0.96	0.64/0.69	0.77	−0.49
15	MFC-PexShort/DJ	**RCGNKRFRWHW**- GIGKLKKAKKFGKKILKK	0.86/H	0.99/1.00	1.0/0.98	1.0/0.9/1.0	0.99/1.00	0.52/0.64	0.03	−1.19
16	MFC-PexNC/DJ	GIGK-G-**RCGNKRFRWHW**-ILKK	0.83/H	0.99/0.99	0.92/0.98	1.0/0.5/1.0	0.94/0.99	0.61/0.61	0.01	−0.65
17	MG2-I_6_V_9_W_12_T_15_I_17_ [183]/E	GIGKFIHSVKKWGKTFIGEIMNS	0.26	0.97/0.99	1.0/0.85	1.0/0.9/1.0	0.93/0.99	0.55/0.57	0.93	−0.64
18	tLyP-1-MG2- I_6_V_9_W_12_T_15_I_17_ [183]/DJ	**CGNKRTR**- GIGKFIHSVKKWGKTFIGEIMNS	0.33	0.87/1.00	1.0/0.27	1.0/0.9/1.0	0.95/0.97	0.50/0.63	0.78	−0.52
19	KW-MG2-I_6_V_9_W_12_T_15_I_17_ [183]/DJ	**KRKRWHW**- GIGKFIHSVKKWGKTFIGEIMNS	0.46	0.80/1.00	1.0/0.50	1.0/1.0/1.0	0.85/0.98	0.55/0.64	0.57	−0.75
20	WFR_8_ -MG2- I_6_V_9_W_12_T_15_I_17_ [183]/DJ	**RRWFRRRRRR**- GIGKFIHSVKKWGKTFIGEIMNS	0.82/H	0.97/1.00	0.97/0.98	1.0/1.0/1.0	0.91/0.98	0.68/0.63	0.84	−1.08
21	MG2-Q_19_ [184]/E	GIGKFLHSAKKFGKAFVGQIMNS	0.48	0.99/1.00	1.0/0.98	1.0/1.0/1.0	1.00/1.00	0.54/0.58	0.89	−0.50
22	MG2-Q_19_-tLyP-1 [184]/DJ	GIGKFLHSAKKFGKAFVGQIMNS-GG- **CGNKRTR**	0.32	0.93/1.00	0.97/0.96	1.0/1.0/0.9	1.00/0.99	0.51/0.57	0.82	−0.23
23	MG2-Q_19_-KW [184]/DJ	GIGKFLHSAKKFGKAFVGQIMNS-GG- **KRKRWHW**	0.61/L	0.96/1.00	0.99/0.97	1.0/1.0/0.5	0.99/0.99	0.56/0.54	0.72	−0.58
24	MG2-Q_19_-WFR_8_ [184]/DJ	GIGKFLHSAKKFGKAFVGQIMNS-GG- **RRWFRRRRRR**	0.77/L	0.93/1.00	0.94/0.98	1.0/1.0/1.0	0.99/0.99	0.60/0.58	0.83	−0.82
25	Max-TI-MG2/DJ ^&&^	GIAKFLDSAKKFGKKFVKTIMQL	0.31	0.99/1.00	1.0/0.98	0.8/0.9/1.0	1.00/0.98	0.57/0.59	0.97	−0.56
26	Max-TI-MG2-tLyP-1/DJ	GIAKFLDSAKKFGKKFVKTIMQL-GG- **CGNKRTR**	0.44	0.95/1.00	1.0/0.99	1.0/1.0/1.0	1.00/0.87	0.61/0.57	0.98	−0.38
27	RtLyP-1-Max-TI-MG2/DJ	**RCGNKRTR**- GIAKFLDSAKKFGKKFVKTIMQL	0.51/L	0.86/1.00	1.0/0.92	1.0/0.9/1.0	1.00/0.97	0.64/0.63	0.86	−0.41
28	Max-TI-MG2-KW/DJ	GIAKFLDSAKKFGKKFVKTIMQL-GG- **KRKRWHW**	0.55/L	0.98/1.00	1.0/0.98	1.0/1.0/0.5	1.00/0.95	0.69/0.57	0.98	−0.75
29	Max-TI-MG2-WFR_8_/ DJ	GIAKFLDSAKKFGKKFVKTIMQL-GG- **RRWFRRRRRR**	0.77/H	0.97/1.00	0.97/0.98	1.0/1.0/1.0	0.99/0.97	0.64/0.58	0.98	−1.00
30	KAF5879953.1 36–47 MFCA	**RCNRKRFRWQWK**	0.97/H	1.00/0.64	0.86/0.98	0.1/0.6/1.0	0.14/0.75	0.56/0.66	0.01	−0.43
31	tLyp-1-RHP [54]/DJ	**CGNKRTR**-YKWYYRGAA	0.78/H	0.21/0.57	0.94/0.96	0.8/0.2/0.7	0.88/0.21	0.47/0.62	0.01	0.07
32	R-tLyP-1-RHP/DJ	**RCGNKRTR**-YKWYYRGAA	0.83/L	0.76/0.65	0.87/0.98	1.0/0.3/0.9	0.89/0.14	0.57/0.63	0.01	0.12
33	MFC-RHP/DJ	**RCGNKRFRWHW**-YKWYYRGAA	0.84/H	0.96/0.86	0.96/0.98	1.0/0.7/0.8	0.72/0.46	0.64/0.62	0.04	−0.51
34	MFC2/DJ	**RCGNKRFRWHW**-GG-RRAKWRR	0.97/H	1.00/0.97	0.60/0.98	1.0/0.9/1.0	0.37/0.91	0.64/0.64	0.01	−0.69
**35**	**MFC-PexSa/DJ**	**RCGNKRFRWHW**- GIGKL**L**K**R**KKFGKKILKK	0.90/H	0.99/1.00	1.0/0.97	1.0/1.0/0.5	0.99/1.00	0.58/0.65/1.60059	0.054	−1.34
36	MFC2-analog/DJ	**RCGNKRLIWHW**-GG-RRAK**T**RR	0.95/H	0.97/0.93	0.22/0.98	1.0/0.9/0.9	0.50/0.84	0.65/0.64/1.61375	0.005	−0.43
37	MG2-analog/DJ	GIGK**L**L**K**SA**L**KFGKAFVGEIMNS	0.177	0.986/1.0	0.998/0.988	0.982/0.9643/0.926	1.0/0.994	0.6163/0.627/1.76783	0.98	−1.28
38	WFR_8_-MG2-analog/DJ	**RRWFRRRRRR**- GIGK**L**L**K**SA**L**KFGKAFVGEIMNS	0.783/H	0.99/1.0	0.839/0.971	1.0/0.99/1.0	0.96/0.98	0.723/0.646/1.5622	0.952	−1.44
39	CA-MA2 [185]/E	KWKLFKKI-P-KFLHSAKKF	0.895/L	0.997/1.0	1.0/0.98	0.983/0.73/0.56	0.94/0.996	0.62/0.645	0.008	−0.09
40	K6L9 [186]/E	LKLLKKLLKKLLKLL	0.958/H	0.918/1.0	0.996/0.92	0.999/0.927/0.71	0.13/1.0	0.62/0.607	0.907	−1.00
41	PR-39 pig P80054	RRRPRPPYLPRPRPPPFFPPRLPP RIPPGFPPRFPPRFP	0.760/L	1.00/1.0	0.993/0.92	1.0/0.857/0.064	0.82/0.965	0.50/0.550	ND	−0.71
42	Pyrrhocoricin [187]/E	VDKGSYLPRPTPPRPIYNRN	0.48	0.35/1.0	0.12/0.576	0.248/0.175/0.064	0.20/0.965	0.481/0.488	0.004	−1.25
43	R_8_FW-Pyrrhocoricin/DJ	**RRWFRRRRRR**- GVDKGSYLPRPTPPRPIYNRN	0.864/L	0.964/1.0	0.41/0.98	1.0/0.61/0.984	0.80/0.96	0.561/0.588	0.064	−1.31
44	PR-35/E	RRRPRPPYLPRPRPPPFFPPRLPPRIPPGFPPRFP	0.762/L	1.0/1.0	0.978/0.9198	1.0/0.805/0.0	0.81/0.925	0.512/0.55	0.001	−0.66
**45**	**PR-35-analog/DJ**	RRRVRPPYLPR**V**RP**Q**PFFP**L**RL**LK**RISPGFPPRFP	0.821/L	0.993/0.995	0.481/0.854	1.0/0.984/0.962	0.90/0.919	0.637/0.581/2.18407	0.012	−1.44
46	CA-MA2-analog1/DJ	KWKLFKKI**L**K**L**LHS**V**KKF	0.895/H	0.996/1.0	1.0/0.9786	0.999/0.875/0.184	0.96/1.0	0.6326/0.735/1.8861	0.848	−0.84
47	L-K6V1-temp [112]- revP9 [188]/DJ	IKKIVSKIKKLLK-**PPWWRRRRR**	0.972/H	0.984/1.0	0.956/0.983	0.998/0.953/0.828	0.75/1.0	0.591/0.664	0.017	−1.17
**48**	**L-K4V1-temp-revP9-** **analog/DJ**	IKKIVS**L**I**L**KLLK-**LPWWRRRRR**	0.959/H	0.999/1.0	0.451/0.982	0.999/0.959/1.0	0.65/1.0	0.74/0.764/1.965	0.190	−1.30
49	CA-MA2-analog2/DJ	KW**R**LFKKI-P-**R**FL**R**SA**RR**F	0.954/H	1.0/0.948	0.977/0.980	1.0/0.935/0.758	0.87/0.992	0.605/0.625	0.054	−1.11
50	Sub-5 [189]/E	**RRWKIVVIRWRR**	0.932/H	1.0/0.994	0.935/0.975	0.784/0.579/0.762	0.30/1.0	0.516/0.643	0.037	−0.76
51	Sub-5-G-nuclear- loc.-signal [190]/DJ	**RRWKIVVIRWRR-G-**PKKKRKV	0.973/H	1.0/0.999	0.494/0.984	0.999/0.930/0.998	0.57/0.993	0.656/0.603	0.007	−0.93
52	**temp V_1_R_9_ (analog-3)-Sub-5/DJ**	**V**KKIVSKI**R**KLLK-GG- **RRWKIVVIRWRR**	0.940/H	0.983/1.0	0.972/0.976	0.999/0.974/0.916	0.92/1.0	0.528/0.641/1.49473	0.098	−0.94

* We used the servers listed in Table 2 and applied them in the same order. All peptides are assumed to be amidated at their C-terminal. MF abbreviation stands for multifunctional. Highlighted peptides (bold name) with underlined activity scores are our selection for the designed peptides with the best overall score (see Table 6). Bold sequence segments have predicted or verified CPP activity. Underlined residues are substitutions for increasing anti-inflammatory activity or decreasing peptide toxicity. ^&&^ Repeated applications or our “Mutator” algorithm (http://split4.pmfst.hr/mutator/ (accessed on 7 August 2022); Kamech et al. [46] suggested amino acid substitutions (underlined) for predicted maximal therapeutic index of the magainin analog Max-TI-MG2.

**Table 6:** Ranking of predictions for the best multifunctional peptide constructs with the reward for a predicted negative mean of hemolytic and toxic activity.

Length-Amph-AMP *	Table- Peptide ^#^	CPP	Anti- Microbial ^&^	Anti- Cancer ^&^	Anti- Viral ^&^	Anti- Fung ^&^	Anti- Inflamm. ^$^	Sum/6	Rank ^†^	Hemol. Probab.	Tox. Score	Reward Low tox.	Total Score ^§^	Overall Rank	CPP Part
25-**α**d-**temp V_1_R_9_**	**T3-37**	0.97/H	0.955	0.965	**1.00**	0.975	0.997	0.9869	1	0.130	−1.27	0.570	0.92734	1st	WFS_6_R_7_
31-**α**t**α**d-**temp R_9_R_14_**	**T4-39**	0.92/H	0.983	0.9445	0.972	0.949	1.0412	0.9682	6	0.010	−1.25	0.620	0.91846	2nd	WFA_5_R_7_
25-**α**d-**temp R_9_**	**T3-36**	0.96/H	0.955	0.97	**1.00**	0.98	0.927	0.9670	7	0.180	−1.30	0.560	0.90886	3rd	WFS_6_R_7_
31-**α**t**α**d-**temp** **R_9_R_14_**	**T4-38**	0.94/H	0.993	0.9495	0.985	0.952	0.9368	0.9594	10	0.028	−1.20	0.586	0.90606	4th	**WFR_8_**
22-**α**d-**temp**	**T4-30**	0.95/H	0.99	0.99	0.927	0.96	0.996	0.9688	5	0.070	−1.12	0.525	0.90540	5th	KW
22-αd-**r-o-p** **A_8_I_15_**	**T2-20**	0.987/H	0.977	0.976	0.863	0.906	0.9038	0.9353		0.007	−1.39	0.692	0.90047	6th	tLyP-1
29-βαd-**PexS**	**T5-35**	0.90/H	0.995	0.985	0.83	0.995	0.9435	0.9414	21	0.054	−1.34	0.643	0.89877	7th	MFC
25-**α**d-**temp**	**T3-9**	0.97/H	0.99	0.975	**1.00**	0.985	0.757	0.9461	19	0.170	−1.35	0.590	0.89523	8th	WFR_8_
31-**α**t**α**d-**temp** **R_9_R_14_**	**T4-37**	0.95/H	0.9985	0.961	0.927	0.98	0.8538	0.9450	20	0.032	−1.18	0.574	0.89200	9th	**WFR_8_**
22-**α**tαd-**temp**	**T5-48**	0.959/H	1.00	0.7156	0.986	0.825	1.1563	0.9472	18	0.190	−1.30	0.555	0.89119	10th	rP9a
31-**α**tαd-**mapegi**n-a2	**T3-50**	0.881/L	0.756	0.8295	0.967	0.983	0.9556	0.8953		0.077	−1.81	0.867	0.89118	11th	TATa
25-**α**d-**BMAP**	**T4-33**	0.905/H	0.991	0.9715	0.993	0.905	0.9868	0.9567	12	0.080	−1.05	0.485	0.88931	12th	KW
35-βtαd- **PR-35a**	**T5-45**	0.821/L	0.994	0.6675	0.982	0.910	1.134	0.9180		0.012	−1.44	0.714	0.88886	13th	whole
25-**α**d- **temp L_9_V_14_**	**T3-35**	0.96/H	0.999	0.97	**1.00**	0.98	0.978	0.9738	4	0.760	−1.47	0.355	0.88540	14th	WFR_8_
28-**α**d-**T2R1-L_7_S_17_-a2**	**T4-32**	0.93/H	0.914	0.974	0.997	0.785	0.9557	0.9260		0.050	−1.30	0.625	0.88300	15th	KT_2_W
25-αd-**BMAP-18**	**T4-25**	0.90/H	1.00	0.98	1.00	0.930	0.875	0.9475	17	0.090	−1.01	0.460	0.87787	16th	KW
**27-αtβ** **d** **-temp V_1_R_9_**	**T5-52**	0.940/H	0.992	0.974	0.963	0.960	0.888	0.9528	14	0.098	−0.94	0.421	0.87684	17th	Sub 5
25-β**α**d-**BMAP-a2**	**T4-36**	0.849/H	0.987	0.9665	0.990	0.912	1.0828	0.9646	8	0.399	−1.09	0.346	0.87616	18th	KW
33-**α**d-**adepantin-1A**	**T4-18**	0.86/L	**1.00**	0.955	**1.00**	0.98	0.8668	0.9436		0.630	−1.56	0.465	0.87525	19th	WFR_8_
25-**α**t**β**d-**novispirin-a1**	**T4-50**	0.903/H	0.959	0.9885	0.920	0.975	0.5942	0.8899		0.195	−1.63	0.7175	0.87307	20th	KW
33-**α**d adepantin-1A-L_15_	T4-51	0.878/L	0.9995	0.9358	0.965	0.972	0.954	0.9506	16	0.826	−1.63	0.402	0.87225	21	WFR_8_
22-r-o-pA_8_I_15_	T2-21	0.985/H	0.98	0.962	0.683	0.91	0.960	0.9134		0.006	−1.22	0.607	0.86963	22	tLyPA_3_-1
20-**α**β BP100	T4-34	0.927/H	0.9975	0.9735	0.987	0.935	1.0426	0.9772	3	0.894	−1.32	0.213	0.86803	23	KW
19-**α**β BP33	T4-9	0.93/H	**1.00**	0.985	**1.00**	0.96	1.001	0.9793	2	0.820	−1.19	0.185	0.86583	25	KW
28-**α**t**α**-novispirin-a1	T3-38	0.94/H	**1.00**	0.8745	0.995	0.978	0.9305	0.9526	15	0.551	−1.17	0.310	0.86073	26	WFL_5_R_7_
35-**α** adep1a	T4-17	0.80/L	0.995	0.965	**1.00**	0.99	0.8552	0.9342		0.660	−1.46	0.400	0.85789	29	WFR_8_
31-**α**tα mapegin-a1	T3-49	0.958/H	0.990	0.798	0.868	0.97	1.1887	0.9621	9	0.717	−1.09	0.187	0.85127	30	TAT
18-**αtα** r-o-p-analog	T2-22	0.991/H	1.00	0.962	0.97	0.795	0.61	0.8879		0.003	−1.26	0.629	0.85091	31	whole
25-**α**-BMAP2-18	T4-52	0.961/H	0.9995	0.988	0.948	0.955	0.6315	0.9139		0.176	−1.09	0.457	0.84862	32	KW
34-**αtα**-Zyk1a	T4-35	0.833/L	0.9985	0.9815	0.983	0.987	0.9377	0.9534	13	0.938	−1.33	0.196	0.84520	33	WFR_8_

* The amphiphilic character of the peptide was assessed by the SPLIT 3.5 server (http://split.djpept.com/split/ accessed on 7 August 2022 [42]). Bold or normal font α, β, and t symbols are stronger or weaker predicted profiles of hydrophobic moments for helix, beta-strand, and turn secondary structure. The “d” symbol is for predominantly disordered structure when indicated by the flDPnn server [191] for the first 20 peptides. The same server predicts DNA and RNA binding sites for all 20 best peptides from 41% to 100% of their residues. Peptide’s abbreviations are in Table 2, Table 3, Table 4 and Table 5. For instance, the temp abbreviation stands for the L-K6V1 temporin 1CEb with the sequence IKKIVSKIKKLLK [112]. The a1 or a2 abbreviation is for analog1 or analog2. The single code letter with the subscript for the residue sequence position is used for substituted amino acids. In the asparagutin case (WFR_8_), R_7_ or R_8_ means the total number of arginines. ^#^ The peptide code number is “Tn-m” for “n” = 2,3,4,5, referring to the corresponding Table, and “m” for the peptide number in Table n. ^&^ Mean values of predicted probabilities for antimicrobial, anticancer, antiviral, and antifungal activity. See Table 2 for server addresses and corresponding references. We used the gray background to highlight cases among 20 best peptides when the probability for anticancer and antiviral activity is close to 1.0 (>0.95). ^$^ Mean value of predicted scores by AIPpred, PreAIP, and AntiInflam servers. See Table 2 for server addresses and corresponding references. The AntiInflam server was included in the calculated mean for the cases when three or fewer amino acid substitutions were enough to raise the predicted score above 1.0 (except for the PR-35 analog with seven substitutions). ^†^ Peptides are first ranked (yellow background) regardless of their predicted hemolytic activity and toxicity. ^§^ Total score is calculated as: (CPP probability + mean antimicrobial probability + mean anti-cancer probability + mean antiviral probability + mean antifungal probability + mean anti-inflammatory score)/6 − (hemolytic activity probability + toxicity score)/2. The subtracted number is a positive reward for low toxicity. We used the blue and green background to rank the 20 best peptides according to their total score.

## 4. Design of Cell-Penetrating Multifunctional Peptides

### 4.1. Advantages of Cell-Penetrating Antimicrobial Peptides

Conventional antibiotics often have difficulties reaching pathogens in mammalian cells. The challenge of eliminating intracellular pathogens reflects in the persistence of related diseases, rising antibiotic resistance, and severe side effects [192,193]. Fortunately, many different drug delivery systems have been developed in recent years. One such delivery mechanism is covalently connecting a bioactive molecule to some cell-penetrating peptide that can target specific cell types, malignant cells, or intracellular pathogens [54]. In this chapter, we shall consider peptide–CPP hybrids. Noninvasive applications of therapeutic peptides conjugated to CPP offer new solutions to the problem of how to overcome the barriers in a body such as the plasma membrane, blood–brain barrier, intestinal lumen, skin barrier, air–lung barrier, blood–lung barrier, nasal cavity, or the posterior segment of the eye [194]. The CPP choice must consider the cell-penetrating ability or probability, uptake efficiency, toxicity, stability, half-life, immunogenicity, and other features that can all change depending on the attached cargo molecule. A short-length CPP conjugate has the practical advantage of being less expensive for synthesis and testing. For a peptide as bioactive cargo, we mainly chose among known antimicrobial or anticancer peptides. Homing peptides are a good choice for targeting specific populations of cells or intracellular organelles.

Peptide–CPP hybrids designed by other authors and us are in Table 3, Table 4 and Table 5. Our primary design goal was to have a broad spectrum of highly predicted functional activities (cell-penetrating, antibacterial, anticancer, antiviral, antifungal, and anti-inflammatory) and as low toxicity as possible. The short conjugate length was the secondary goal because combining many different functions in a short hybrid peptide is difficult.

### 4.2. Potential for Clearing Intracellular Drug-Resistant Bacteria

Besides cancer cells as targets for CPP-cargo molecules, there is a pressing need to discover nontoxic last-resort drugs to eliminate intracellular multidrug or pan-resistant bacteria [195]. Colistin is a peptide-fatty acid conjugate that belongs to the last-resort class of antibiotics against hard-to-treat bacteria. For several decades it was abandoned in medical practice due to its nephrotoxicity. Its toxicity and additional resistance induction are obstacles to clinical usage [196,197]. After multidrug resistance proliferated, medical doctors are again treating endangered patients with colistin by carefully balancing positives (saving patient’s life) and negatives (a certain degree of damage to some organs).

It would be better to widen the availability of nontoxic peptides capable of clearing resistant intracellular bacterial targets [198]. Fortunately, some bacteriocins are highly specific bactericides for their target bacteria and nontoxic to eukaryotic cells. Among them, peptidoglycan hydrolases induce bacterial lysis by cleaving specific conserved bonds within the peptidoglycan (PG) of the bacterial cell wall. PG target bonds are well conserved, making it difficult for bacteria to develop resistance against PG hydrolases. These advantages are enhanced when PG hydrolases are fused to penetratin or some other cell-penetrating peptide. Such constructs eradicate intracellular drug-resistant *Staphylococcus aureus* [199]. These authors used the bacteriocin enzyme lysostaphin fused to penetratin or TAT peptide from HIV. Both constructs were equally efficient in clearing intracellular antibiotic-resistant strains of *S. aureus* responsible for recurrent infections. Therefore, CPP-fused PG hydrolases are promising therapeutic applications of penetratin and other cell-penetrating peptides.

Some cationic antimicrobial peptides (AMPs) are selective and refractory to resistance mechanisms developed by microbial pathogens and cancer cells [171]. Ribosomally synthesized peptides are more costly than small molecular weight drugs but less expensive compared to recently developed immunotherapy. As host defense peptides, AMPs are an essential component of our immune system, with some able to translocate across membranes without the need to design artificial AMP–CPP hybrids. There should be no undesired immune response to peptides recognized as innate by the human body, even if some slight modifications are introduced to enhance their stability.

Unfortunately, the research about AMPs is underfunded by pharmaceutical companies and governmental agencies charged with supporting health-oriented innovations. There was an initial failure of AMPs to achieve clinical applications, which resulted in a widespread bias against them, despite all evidence that AMPs can be used as multifunctional agents effective against bacteria, fungi, viruses, drug-resistant biofilms, and cancer [200,201,202,203,204,205]. Nevertheless, the promise of multifunctional AMPs will eventually come to fruition [206].

### 4.3. Short Cell-Penetrating Peptides and Their Conjugates

Optimized penetratin analog RKKRWFRRRRPKWKK [56] has six arginines, five lysines, and two tryptophans. Besides its high cell-penetrating ability, *in silico* predictions make a case for antibacterial, anticancer, and antiviral activity with considerably lower hemolytic activity than the pAnp penetratin (see prediction results for peptide 16 from Table 2). In known homeoproteins, there is no natural penetratin-like peptide of similar length (15–16 residues) with such a large number of positive charges (≥+10). However, the hypothetical protein OLQ14316.1 from coral dinoflagellate symbiont *Symbiodinium microadriaticum* [207] contains a similar sequence R(603)RRRRRWFRRRRRRWFRKI(621), named DiR_6_WF (Table 3, peptide 2), with an even higher number of arginines.

The decapeptide RRWFRRRRRR (abbreviation WFR_8_) from that domain has the best chance of being a short CPP peptide, according to the CellPPD server [167]. Both peptides have a high CPP probability (0.99) and are predicted as nontoxic with antimicrobial, antiviral, and anticancer activity (see prediction results for peptides 2 and 3 from Table 3). Identical decapeptide R(122)RWFRRRRRR(131) from the asparagus plant (*Asparagus officinalis*) uncharacterized protein A0A5P1FK94 with 142 residues is also the best predicted CPP in that protein. We shall name it asparagutin. The natural function of asparagutin is unknown. The WF doublet from asparagutin is conserved in all penetratin-like peptides from homeodomains (see Table 1).

In Table 3, we mostly use pAntp penetratin and short CPP candidates—the decapeptide RRWFRRRRRR and its reversed version RRRRRRFWRR (peptides 3 and 4 from Table 3), which to our knowledge, have never been synthesized and tested. Asparagutin is considerably shorter than penetratin, but it may be more difficult for solid-state synthesis. Wender et al. [208] proposed a better pathway for synthesizing polyarginine peptides. We assume that difficulties synthesizing the RRWFRRRRRR sequence or its reversed analog should no longer be a serious issue. According to the VaxiJen server by Doytchinova and Flower [209] for the immunogenicity prediction (http://www.ddg-pharmfac.net/vaxijen/VaxiJen/VaxiJen.html, (accessed on 7 August 2022)), the asparagutin is the probable antigen for parasites and fungi and probable non-antigen for bacterial, viral, and tumor cell targets. The predicted cleavage site for different proteases is after the Phe residue (the result of Song et al. [210] server analysis at the link: https://prosper.erc.monash.edu.au/, (accessed on 7 August 2022)). Six terminal arginines after protease cleavage should still have the CPP ability, with somewhat lesser uptake efficiency than the widely used eight arginine CPP [211]. The hemolytic activity is negligible for the reversed sequence RRRRRRFWRR (0.08 probability).

Wei et al. [168] used molecular simulations to design the KRKRWHW peptide (named KW), which exhibited little cytotoxicity and high penetrating efficiency into mammalian cells. For that peptide and its 30 conjugates (see Table 4 peptides 1–11, 14–16, 19, 25–28, 30, 33, 34, 36, 40, 50, and 52 and Table 5 peptides 8, 19, 23, and 28), we obtained variable predictions for the hemolytic activity. Due to the importance given to low toxicity estimates, five KW-containing peptides with a low probability of harming red blood cells (0.4 or lesser probability) and low toxicity score (−1.01 or less) entered among the 20 best multifunctional constructs with a high overall score (see Table 6). These are hybrid peptides 25, 30, 33, 36, and 50 from Table 4. Despite different bioactive cargo (temporin, novispirin, or BMAP antimicrobial peptides), an excellent multifunctional activity is possible for all of them.

Identical septapeptide KRKRWHW is present in the C-terminal segment GQEQR**KRKRWHW**RKFHKK of bacterial protein A0A1G1FKX2 from Nitrospiraceae bacterium named the PSP1 C-terminal domain-containing protein (preliminary data). The segment is also predicted with a high uptake efficiency (CPP probability of 0.91) and increased antibacterial and antifungal activity compared to its KRKRWHW fragment. Its binding affinity for bacterial or eukaryotic mRNA may be more important according to the PROSITE pattern https://prosite.expasy.org/doc/PS51411 (accessed on 7 August 2022) for the PSP1 C-terminal domain profile. The DP-Bind server predicts DNA-binding sites for all but the first three residues: QRKRKRWHWRKFHKK. When the whole A0A1G1FKX2 protein (preliminary data) is examined with the RNABindRPlus web server http://ailab1.ist.psu.edu/RNABindRPlus/ (accessed on 7 August 2022), thirty binding sites to RNA are predicted, but none of them are even close to the C-terminal sequence GQEQR**KRKRWHW**RKFHKK.

The biological significance of the PSP1 C-terminal domain for cell cycle regulation is still under investigation [212]. Anyway, it is possible that rationally optimized molecular docking and dynamics simulations by Wei et al. [168] rediscovered short nontoxic CPP, which nature has already developed as a protein motif in some bacteria. The KRKRWHW peptide (KW) exhibits non-covalent binding to disaccharide trehalose. Trehalose provides an exceptional stabilization of proteins during the desiccation procedure for extended storage [213,214]. Loading trehalose in mammalian cells is considerably more efficient in combination with the KW peptide and less damaging than other procedures for introducing that disaccharide into cells [168].

Anticancer and antiviral activities are well predicted for the KW peptide fused to BMAP-18 cathelicidin fragment GGLRSLGRKILRAWKKYG of BMAP-28 antimicrobial peptide, which targets mitochondria [174] (peptide 25, Table 4). BMAP antibiotics cause mitochondrial depolarization and cytochrome c release by opening the mitochondrial permeability transition pore.

We used peptides CGIKRTK, CGAKRTK, CGNKRTR, RCGNKRTR, and RCGIKRTK as short CPPs for designing multifunctional constructs (see Table 2 peptides 15, 18, 20, and 21; Table 3 peptide 8; Table 4 peptide 20; Table 5 peptides 1, 2, 7, 18, 22, 26, 27, 31, and 32). The tLyP-1 tumor-homing peptide CGNKRTR [146] is found in predicted helicases from *Ferroplasma* species (Archaea) HII82410.1, A0A1V0N279, and A0A7K4FM37. *Ferroplasma* sp. loves a hot acid, heavy-metal rich environment (pH from 0 to 2 and temperatures from 35 to 55 °C. The archeon exhibits strange ancient bioenergetics dependent on oxidizing ferrous iron (Fe^2+^) to ferric iron (Fe^3+^). Helicases containing the CGNKRTR motif from *Ferroplasma* sp. are classified as DEAD/DEAH-box helicases—the essential enzymes for the survival of advanced invasive melanomas [215], lung adenocarcinoma [216], and renal cell carcinoma [217]. Hence, a connection may exist spanning billions of years of biological evolution with the evolution of invasive cancer cells.

Unsurprisingly, helicases have been popular study subjects from 1976 onward due to their ability to unwind duplex DNA [218]. The CGNKRTR peptide is also present in the unchanged or slightly changed form at the C-terminal of integral membrane protein for sodium-dependent phosphate transport from *Actinia tenebrosa* and *Nematostella vectensis* (sea anemones): respectively, XP_031563687.1, and XP_032222729.1 (A7RG57). Septapeptides are too short of having solid evidence about their biological significance in the absence of broad conservation. Octapeptide RCGIKRTK from the C-terminal of *N. vectensis* predicted protein A7RG57 has higher probabilities for multifunctional activity than CGNKRTR (see peptide 2 prediction results in Table 5). All conjugates mentioned above with the CGNKRTR or its analogs are interesting for synthesis and testing. All have a well-predicted broad activity spectrum, and only two (peptides 26 and 27 from Table 5) have higher predicted toxicity to healthy mammalian red blood cells than magainin-2.

The predicted probability for anticancer activity is high for some hybrid peptides. It is 0.92 or higher as the output of both ACP servers for peptides 20 and 21 from Table 2, 20 from Table 4, and peptides 2, 7, 22, 26, 27, and 31 from Table 5 containing tLyP-1 or its analogs. The IFLLWQR septapeptide (IF7, see peptide 13 from Table 4) binds to the annexin-1 protein, which is over-expressed on the endothelial caveolae surfaces of different tumors [219]. Through endocytosis, annexin family proteins are internalized, allowing IF7 conjugates with anticancer drugs (such as anticancer peptides) to penetrate tumor cells freely. Many other short tumor-homing peptides are described in the literature [170].

Xia Xu developed with collaborators several additional short CPP for helping anticancer drugs enter tumor cells. These are RRRRRWW [220], RRRRQWWQW [221], and RRRRRWWPP [188]. Employed servers suggest an antibacterial, antiviral, and antifungal activity for the IKKIVSKIKKLLK-PPWWRRRRR conjugate, good cell-penetrating ability, and low toxicity (see peptide 47, Table 5). The reversed sequence of the RRRRRWWPP positioned the proline residues near the peptide middle due to expectations of increased selectivity [185,222].

The high electric field of energized mitochondria attracts arginine-rich CPPs after they pass through the plasma membrane. Peptide 13 from Table 4 may have multiple means for internalizing tumor cells and reaching mitochondria due to its asparagutin moiety. Peptide 6 from Table 3 is an example of how attached asparagutin RRWFRRRRRR can promote the uptake of mitochondrial-homing peptide MIASHLLAYFFTELN (dubbed pHK). Woldetsadik et al. [147] fused the homing peptide with the penetration-accelerating sequence GKPILFF [223]. The hybrid peptide MIASHLLAYFFTELN-GKPILFF-amide (pHK-PAS) disrupted the association of hexokinase II (HK2) with mitochondria in cancer cells. It led to mitochondrial dysfunction and apoptosis of cancer cells without substantially increased cytotoxicity to normal cells [147]. Thus, the hybrid peptide containing pHK and either RRWFRRRRRR or GKPILFF can be the artificial death signal for malignant mitochondria with potential therapeutic applications (see peptide 6, Table 3). The pHK-PAS peptide is predicted as non-ACP by both servers for anticancer peptides illustrating difficulties in constructing such servers.

Malignant mitochondria and their protein–protein interactions contributing to cancer phenotype are key targets for chemotherapy because the respiratory metabolism of mitochondria is crucial for cancer survival despite the Warburg effect. Mitochondrial structure and function are different between normal cells and cancer cells. These differences offer a potential for the design of anticancer compounds acting on mitochondria for the selective killing of cancer cells [224]. The peptide pHK prevents the hexokinase II association with outer mitochondrial membrane VDAC porin [225]. The pentadecapeptide M(1)IASHLLAYFFTELN(15) is the VDAC-binding N-terminal domain of human HK2 (Uniprot entry P52789), acting as a surrogate peptide for HK2. HK2-VDAC association helps keep mitochondrial permeability transition pores in closed conformation when bound to the ATP–synthasome complex [226]. Mitochondria die together with the cell containing mitochondria when transition pores are continuously open due to the inhibition of the HK2-VDAC association. HK2 enzymes are gatekeepers of life and death [227].

There are, of course, many other possibilities to fuse the pHK peptide with some cell-penetrating peptide for easier access to malignant mitochondria. One such option for targeting cancer cells with a designed artificial death signal has been explored by Chiara et al. [225]. These authors used the HIV-1 TAT CPP peptide to create the MIASHLLAYFFTELN(β-Ala)-GYGRKKRRQRRRG-amide hybrid, called HK2-TAT. Unfortunately, subsequent experiments revealed that a low concentration of that hybrid peptide (1 μM HK2-TAT) causes rat heart ischemia [228]. Hence, additional study is needed with different pHK-CPP conjugates. One possibility is the MIASHLLAYFFTELN-GG-RCGNKRTK construct that uses the tLyp-1 analog for the penetration acceleration of pHK. Its advantage would be considerably lower toxicity (0.09 probability for hemolytic activity) in comparison with HK2-asparagutin (0.44), HK2-TAT (0.34), and HK2-PAS (0.29).

Designed short tumor-homing peptides KW and tLyP-1 (peptide 1 from Table 4 and peptide 1 from Table 5) are similar in N-terminal and C-terminal parts. The hybrid construct CGNKRFRWHW may have a good combination of CPP and other multifunctional activities for its short length. We added the Arg residue at its N-terminal because it is present as a natural tLyP-1 analog RCGIKRTK. Central KRFR motif is present in some cathelicidin antimicrobial peptides. The resulting RCGNKRFRWHW conjugate (peptide 3 from Table 5) will be named MFC for the Multi-Functional Construct. A likely membrane-stabilized structure of the MFC is an amphipathic beta-strand for residues 5–11 (SPLIT prediction). The DP-Bind server predicts DNA binding for all RCGNKRFRWHW residues. The most interesting expected features are low toxicity and the absence of any hemolysis combined with high cell-penetrating, anticancer, and antiviral activity of that undecapeptide. Two C-terminal tryptophans are natural fluorescence probes for examining the location and microenvironment of MFC added to membrane vesicles, organelles, or living cells. A high density of positive charges and hydrophobic residues should help MFC accumulation by topologically closed membranes with active bioenergetics. Histidine presence should make it sensitive to pH changes. The presence of reactive cysteine facilitates chemical modification for fine-tuning desired effects.

BLASTP search discovered only one natural MFC analog (peptide 30 from Table 5 named MFCA) with a similar sequence RCNRKRFRWQWK. The MFCA peptide is found as the 36–47 segment of the uncharacterized protein (partial) KAF5879953.1 during a recent genome analysis of walking catfish Clarias magur. Its predicted CPP probability is promising 0.97 with a high score of 0.76 for uptake efficiency, but other predicted multifunctional activities are not enhanced compared to MFC. The equally low likelihood for the hemolytic activity of 0.01 leaves enough space for fine-tuning that peptide without making it toxic to healthy human cells. Hybrid peptides 11, 12, 14–16, and 33–35 from Table 5 illustrate how adding bioactive cargo sequences to MFC can result in widely different hemolytic activity predictions. Seven conjugates are associated with predicted hemolytic activity of 0.06 or less (peptides 12, 15, 16, and 33–35 from Table 5). For three of them (peptides 15, 16, and 35), we used the same design approach as before by adding a shorter pexiganan sequence (PexShort) or pexiganan’s N and C terminal tetrapeptides (PexNC) (see peptides 8–10 from Table 2) to respective MFC terminals.

The peptides 15 and 35 from Table 5 with sequences RCGNKRFRWHW-GIGKLKKAKKFGKKILKK and RCGNKRFRWHW-GIGKL**L**K**R**KKFGKKILKK have a maximal probability (between 0.97 and 1.0) for clearing antibacterial, antifungal, and anticancer intracellular targets. Peptide 35 is optimized for anti-inflammatory activity after two amino acid substitutions (bold and underlined residues), and its overall rank is seventh among all of the considered peptides from Table 2, Table 3, Table 4 and Table 5. An unexpected finding is a high probability (0.93 or higher) for the antifungal activity of MFC conjugates 11, 12, 14–16, and 35. The pexiganan analog cargo of these peptides may have a similar capability of depolarizing mitochondria and killing fungi and parasitic intracellular protozoans as the pexiganan but must be stabilized against proteolytic degradation [229].

For peptide 12 from Table 5, the bioactive cargo is Zp3a sequence GIKAKIGIKIKK (see also peptide 32 from Table 3). That peptide was recently designed by Zeng et al. [161] to eradicate the resistant Vibrio species pathogens, a frequent cause of disease outbreaks related to seafood consumption. When combined with our MFC construct, or asparagutin, a good compromise is achieved for Zp3a hybrids for predicted toxicity absence and broad-spectrum multifunctional activity. These molecules are more likely than Zp3a to enter the cytoplasm and disrupt mitochondrial membranes.

Mitochondrial-targeting peptide KLLNLISKLF is the prodeath domain MTD of the Noxa, the BH3-only Bcl-2 family protein [157,178,230]. It causes cellular death by opening the mitochondrial permeability transition pore and needs some cytosolic factor to become toxic. Moreover, the peptide requires help to penetrate the cytoplasmic membrane to reach mitochondria. Seo et al. [178] used the CPP-MTD sequence RRRRRRRRGRQ-KLLNLISKLF (peptide 29, Table 4) to study MTD killing mechanism. Jeong et al. [157] used the cationic RIMRILRILKLAR segment from the S5 subunit of a voltage-gated potassium channel (Kv2.1) connected to KLLNLISKLFCSGT via glycine triplet. We fused it with the asparagutin (peptide 27, Table 3) or the KRKRWHW CPP sequence (peptide 15, Table 4). All multifunctional predictions are pretty good for these three hybrid peptides. Low toxicity predictions are, however, questionable because all cell types can be penetrated, and the selectivity for cancer cells is not expected without some tumor-homing mechanism.

There are tumor-homing peptides that can be fused to the MTD. Seo et al. [178] used CGNKRTRGC and CNGRCVSGCAGRC tumor vascular-targeting motifs discovered by Arap et al. [231] to design selective MTD–CPP hybrids. The C2Pred server by Tang et al. [23] predicts that the hybrid peptide CGNKRTRGCGGKLLNLISKLF (named TU3: MTD) gains the CPP ability. That was verified in experiments by Seo et al. [178]. The Chosun University from South Korea patented TU3: MTD and similar peptides in 2012 (US patent 2012/0165269 A1).

Pfeiffer et al. [176] discovered that the antimicrobial peptide mastoparan (INLKALAALAKKIL-amide) facilitates the mitochondrial permeability transition. Mastoparan peptide from wasp venom has a broad spectrum of activities. Among others, it causes cell death of malignant melanoma cells by activating the mitochondrial apoptosis pathway [232]. The hybrid peptide KW–mastoparan (peptide 27 from Table 4) has promising multifunctional potential too.

Peptide 24 from Table 4 is the DP1 pro-apoptotic peptide constructed by Mai et al. [173] with the sequence: RRQRRTSKLMKR-GG-KLAKLAKKLAKLAK. The N-terminal half is the protein transduction domain PTD-5 [233], which is connected via Gly-Gly linker to the C-terminal antimicrobial peptide (KLAKLAK)2 [234]. The DP1 is an efficient killer of tumor cells from accessible solid tumors both in vitro and in vivo. The probable mechanism is disrupting the mitochondrial membranes from these cells [173].

### 4.4. Magainin-2 Analogs Fused to Cell-Penetrating Peptides

Our Mutator server for predicting the therapeutic index TI [46] results in the maximal possible TI = 94.9 for the magainin analog GIAKFLDSAKKFGKKFVKTIMQL (peptide 25 from Table 5). We underlined substituted residues regarding magainin-2. Maximal TI is the best compromise between low hemolytic and robust antimicrobial activity. That magainin analog entered before or after CGNKRTR CPP into constructs 26 and 27, which we designed for the present paper. The HAPPENN server by Timmons and Hewage [40] rejects both magainin conjugates after a probability prediction of 0.98 and 0.86 for their hemolytic activity. It illustrates how different algorithms for predicting the same functionality can produce contrasting results.

Some examples when predictions agree with experimental results are magainin-2-pAntp [172] and magainin-2-bombesin conjugate [171,235] (see prediction results for peptides 22 and 23 from Table 4). Magainin-2 and bombesin were both isolated from frog skin. Bombesin is a cancer-homing peptide apt to recognize various human cancer cells. The magainins exhibit a modest anticancer activity (see peptide 6 from Table 5 and references [236,237,238]. Liu et al. [235] provided a positive answer to whether the conjugation of magainin 2 (MG2) to the bombesin could enhance the selectivity and cytotoxicity of hybrid peptide MG2B against tumor cells. It induced apoptosis of tumor cells in vivo and in vitro. The killing mechanism involves increased binding to cancer cell membranes and increased translocation into these cells. Cellular uptake of MG2B was confirmed by Liu et al. [235] after using fluorescein-labeled MG2B and fluorescence-activated cell sorting. Hence, we have the experimental confirmation for the CPP activity of MG2B despite Table 4 (peptide 22) prediction of the smallest CPP probability (0.30) for MG2B among all 52 peptides from that table. Unconfirmed MG2B ability is for treating polymicrobial co-infections (bacterial, viral, and fungal) and cancer. Immunocompromised persons receiving common anticancer drugs, patients with organ transplants exposed to immunosuppressants, or patients with a partially destroyed immune system (after HIV infection, for instance) are prone to co-infections. They can benefit from antimicrobial peptide conjugates with the unique potential to fight such infections [171].

Liu et al. [172] also examined magainin-2-penetratin conjugate (MG2A abbreviation, peptide 23 from Table 4) for its selective anticancer activity. They observed that penetratin binds to chondroitin sulfate (CS), which is overexpressed on the surface of some tumor cells. Thus, penetratin should be able to act as a tumor-homing and cell-penetrating peptide at the same time while enhancing the anticancer activity of magainin 2. Achieved selectivity was not outstanding because the therapeutic index was not higher than three to five, meaning that cytotoxicity to normal cells was only five times lower. Still, MG2A performed better than MG2B, according to predictions for all beneficial activities (Table 4). Liu et al. [172,235] did not examine these peptides’ antiviral and antifungal efficacy.

Magainin analogs coupled to shorter CPP are in Table 5 (peptides 7–9, 18–20, 22–24, 26–29, and 38). Some of them have better predicted overall performance than MG2A. In the absence of experimental confirmation, there is no way to ensure their therapeutic index is also better, but we have some reasons to expect so. Tumor-homing peptide CGNKRTR and other short CPPs, such as KRKRWHW, RCGIKRTK, RCGNKRFRWHW, RRWFRRRRRR, and RRRRRRFWRR may be able to provide good selectivity. Little cytotoxicity to mammalian cells and high penetrating efficiency was confirmed for the KRKRWHW peptide [168] (peptide 1 from Table 4). However, the predicted hemolytic activity for hybrids 7–9, 18–20, 22–24, 26–29, and 38 is spread around the probability for magainin 2 (0.83) with no value lower than 0.57 for peptide 19 (the conjugate with KRKRWHW).

One can find in the literature multiple confirmations for the broad-spectrum activity of magainin 2, its analogs and hybrids. It includes antibacterial [182,239], antiviral [240], antiprotozoal [241], and antifungal activity [242] in addition to antitumoral properties. To lower production costs, recombinant expressing systems have been developed to obtain large amounts of biologically active peptides [239]. Certain magainin analogs from Table 5 also have confirmed antimicrobial activity (peptides 10 and 13, [182]; peptide 17 [183]; peptide 21 [184]). Peptides 10 (9P0-1) and 13 (9P1-3) exhibited, respectively, 8 to 125 and 4 to 65 times stronger antibacterial activity than their parent peptide 6 (magainin-2) in Azuma et al. [182] experiments with *Escherichia coli* ATCC25922 and *Staphylococcus epidermidis* ATCC12228 strain. That would be difficult to anticipate based on a slight probability increase (from 0.95 to 0.99) for antimicrobial activity of analogs 10 and 13 by the CAMP_R3_ algorithms (the SVM module) reported in Table 5. The CAMP_R3_ Discriminant Analysis (DA) classifier obtains the same (correct) ranking for the antimicrobial potency, that is, 9P0-1 > 9P1-3 > MG2.

Older designed MG2 analogs are peptide 17 [183] and peptide 21 [184] from Table 5. Predicted SVM probabilities by the CAMP_R3_ server are 0.965 and 0.985 for the antimicrobial activity of these peptides. The peptide 17 has confirmed antibacterial potency is from 6 to 40 times more potent in comparison to MG2 against, respectively, *Pseudomonas aeruginosa* and *Escherichia coli*. A slight increase from 0.946 (for MG2) to 0.965 (for peptide 17) for the probability of AMP activity cannot be easily interpreted as confirmation of the server’s accuracy in predicting an order of magnitude stronger antibacterial activity detected in experiments. Instead, it is a possible indication that the applied design principles of Dathe et al. [183] are a good choice. For peptide 21, one amino acid substitution (Q19) was enough for Matsuzaki et al. [184] to observe 4 to 8 times stronger antibacterial activity against the *Acinetobacter calcoaceticus* ATCC 14987 and *Escherichia coli* ATCC 8739 strains. That significant improvement also corresponded to a slight increase in predicted SVM probability, from 0.946 for MG2 to 0.985 for Q19MG2. Attached asparagutin to peptide 17 significantly increased the probability for the CPP activity of the hybrid peptide 20 (also from Table 5) without any apparent decrease in its potential for other MF activities. Two CPP hybrids with peptide 21 with similar predicted features are peptides 23 and 24.

### 4.5. Imperfect and Perfect Activity-Enhancing Palindromes

The palindromic motifs RLLRRLLR and RWQWR enhance the antibacterial activity against Gram-negative and Gram-positive strains [243] when chimeric peptides are constructed based on buforin 2 sequence TRSSRAGLQFPVGRVHRLLRK [159] and lactoferricin fragment RRWQWRMKKLG [244]. Both buforin 2 and lactoferricin have confirmed strong antibacterial, anticancer, antifungal, anti-endotoxin, DNA-binding, and cell-penetrating properties (see [8,159,245,246,247] for validated activities of buforin-like peptides, and [248,249,250,251] for lactoferricin-like peptides). Those and similar palindromic motifs can be employed as LEGO pieces to achieve the desired fine-tuning of desired specificity and selectivity. Asparagutin decapeptides RRWFRRRRRR and RRRRRFWRR are imperfect arginine-rich palindromes with an excellent CPP potential (peptides 3 and 4 from Table 3).

In silico tests were performed with 48 asparagutin hybrids, including some analogs with one amino acid substitution, which decreased the number of arginines to seven. These are peptides 3, 4, 6, 7, 9–16, 20–29, 31–33, 35–38 and 42 from Table 3, peptides 12, 13, 17, 18, 21, 35, 37–39, 41–45, and 51 from Table 4, and peptides 9, 20, 24, 29, 38, and 43 from Table 5. Summary Table 6 lists 8 asparagutin hybrids among the best 20 multifunctional peptides according to the overall score. All magainin analogs fused to asparagutin retained the hemolytic activity and toxicity predictions similar to or worse than magainins. That eliminated them from the ranks of the 20 best peptides (Table 6) due to the strict requirements of the overall score for significantly lower hemolytic activity and toxicity predictions.

Some authors concluded that the guanidino groups from arginines play a crucial role in the membrane permeability of various molecules having different structures [211,252]. Designed penetratin analogs underlined the importance of the cell-penetrating role of the last seven residues of *Drosophila* pAntp penetratin [253,254], namely, residues R(10)RMKWKK(16). It is the motif BBXBXBB when B stands for cationic residues (R, K) and X stands for hydrophobic residues. Alanine substitutions at each sequence position of that septapeptide destroyed the cell-penetrating function of penetratin analogs except for position 12 (Met-12 to Ala-12 substitution). Table 1 illustrates that natural evolution during the last billion years also tolerated alanine substitution at the twelfth position of all penetratin analogs. Examples of penetratin-like peptides from all animals (including sponges and Placozoa) contain the same BBXBXBB palindromic motif. Exceptions from that septapeptide palindromic rule are easier to find in homeotic proteins from other kingdoms of life. Degenerate peptidic palindrome would probably be a better description [255] because palindromic BB sides are connected with an asymmetric linker region (XBX is usually MKW or AKW).

Binding to palindromic DNA sequences with perfect dyad symmetry does not require an equally ideal arrangement of the recognition helix from a transcription factor. The DNA-binding proteins often contain imperfect palindromic motifs, which mediate interaction with the DNA palindromic sequence. For instance, the RRSRARK septapeptide from DNA-recognition helix L(230)KRARNTEAARRSRARKLQRMKQL(253) or A(229)LKRARNTEAARRSRARKLQRMKQ(252) [256] of yeast transcriptional activator GCN4 (2DGC PBD identification for the P03069 protein) is anchored inside the major groove of the palindromic ATF/CREB site and conforms to the same BBXBXBB peptide palindrome with an asymmetric linker [257,258].

The BBXB is the simpler of two Cardin–Weintraub motifs [110] for heparin sulfate proteoglycan recognition [259], indicating that penetratin-like peptides can first bind to negatively charged glycosaminoglycans before they enter eukaryotic cells. Most cationic CPP conform to this motif due to the high density of positively charged residues [260]. Cell surface proteoglycans promote the uptake of arginine-rich penetratin-like peptides [261], but the uptake mechanism is still disputed [53,262]. Peptide-phospholipid interaction at the plasma membrane surface may mediate internalization at low, while accumulated peptide-glycosaminoglycan clusters activate endocytosis at higher, peptide concentrations [263]. By the way, both choices for the recognition helix (see above) from the GCN4 master regulator of gene expression (which activates more than 500 genes [264]) also have a high probability (0.95 to 0.96 according to the MLCPP server) to act as cell-penetrating peptides. So does the recognition helix ERKRLRNRLAATKCRKRKLERIAR [256] from the JunB prokaryotic transcription factor (CPP probability 0.96), which contains shorter BBXB and longer BBBXXB CW motifs (underlined). A dual role of CW motifs is essential for exported morphogens such as Sonic hedgehog protein and growth factors midkine and pleiotrophin, which bind to heparan sulfate in the form of monomers or multimers and show bactericidal activity [265,266].

### 4.6. Construction of Chimeras Containing Bacterial Pheromones or Ribosomal-Homing Peptide

Almost all chimeric peptides from Table 3, Table 4 and Table 5 are predicted to exhibit antibacterial, antiviral, and anticancer activity. Homing peptides often gain multifunctional abilities when fused to CPP sequences. Adding the N-terminal ribosomal-homing peptide YKWYYRGAA (RHP) to penetratin produces peptide 5 from Table 3 with the sequence YKWYYRGAARQIKIWFQNRRMKWKK, which readily enters into and kills all eukaryotic cells, whether healthy or malignant [54]. A killing mechanism involves binding to the ribosomal protein RPL29 and disrupting ribosomal function. Both algorithms for predicting anticancer activity, the ACPred [26] and mACPred [27], agree on predicting high ACP probability (respectively, 0.95 and 0.98). Antiviral activity for that peptide is also possible (probabilities equal to or higher than 0.8). In vivo usefulness is doubtful due to the peptide’s nonselective cytotoxicity, which agrees with the probability of 0.97 for its hemolytic activity.

Sequence 7 from Table 3 contains the same ribosomal-homing motif, but its CPP part is our WFR_8_ peptide. Predictions are better for almost all activities calculated in that Table than the peptide 5 results. The most encouraging is the prediction by the HAPPENN server for hemolytic activity. The peptide YKWYYRGAARRWFRRRRRR is expected to be non-hemolytic (with a small probability of 0.12 for the hemolytic activity). The predicted absence of hemolytic activity is even better for peptide 2 from Table 4 (0.02 probability), which we constructed as fused ribosomal-homing peptide YKWYYRGAA and short cell-penetrating sequence KRKRWHW designed by Wei et al. [168]. Hexadecapeptides YKWYYRGAAKRKRWHW and KRKRWHWGYKWYYRGAA (also 0.02 probability for hemolytic activity) look like promising lead compounds for selective anticancer activity (probability range from 0.97 to 0.99). Cell-penetrating peptide-based anticancer therapies provide the advantage of rapid delivery to intracellular targets and low toxicity compared to other drugs [267,268].

We can also consider designed hybrids when ribosomal-homing peptide YKWYYRGAA is fused with other shorter CPPs of minimal toxicity, such as reverse-WFR_8_, CGNKRTR, RCGIKRTK, and RCGNKRFRWHW (respectively, peptides 4 from Table 3, and 1–3 from Table 5). These are sequences YKWYYRGAARRRRRRFWRR (peptide 33 from Table 3), CGNKRTRYKWYYRGAA, RCGIKRTKYKWYYRGAA, and RCGNKRFRWHWYKWYYRGAA (peptides 31–33 from Table 5). All of them should have good cell-penetrating activity (probability range from 0.78 to 0.97) without any hemolytic activity (probability predictions of 0.04 or less). If some other well-predicted activities are confirmed (anticancer, antiviral, or antifungal) among these four MF candidates, this would be an additional motivation for drug development.

The significant achievement in using pheromones for targeting specific pathogenic bacteria is the construction of the C16G2 peptide TFFRLFNRSFTQALGKGGGKNLRIIRKGIHIIKKY, which is specifically targeted toward dental caries causing *Streptococcus mutans* [269,270]. The underlined domains in the peptide’s tripartite structure have different functions. The N-terminal part is the targeting sequence TFFRLFNRSFTQALGK derived from *S. mutans* competence-stimulating peptide, quorum-sensing bacterial pheromone. By itself, this domain has weak antibacterial activity. The GGG triplet is introduced next to provide a flexible linker. Underlined C-terminal domain KNLRIIRKGIHIIKKY is well-known broad-spectrum peptide antibiotic novispirin G10 [152,271] derived from sheep AMP ovispirin-1 by glycine for isoleucine substitution at the sequence position 10 to decrease ovispirin toxicity to human cells. It is the “killing domain” forming kinked amphipathic alpha helix in a membrane with resulting high hydrophobic moment. The HAPPENN and ToxinPred offer conflicting predictions. Expected hemolytic activity is very high (0.986 probability), while toxicity is low (−0.98 score).

Just-described discoveries opened a new field of specifically targeted chimeric antimicrobial peptides with a bright perspective of being used daily as a mouth rinse or as an essential ingredient in toothpaste to prevent caries. The importance of research in the case of C16G2 is illustrated by many clinical NIH-funded trials involving voluntary participants, with seven already completed: https://clinicaltrials.gov/ct2/results?term=C16G2&Search=Search (accessed on 26 July 2022).

One can use the same principle to construct other chimeric antimicrobial peptides with a flexible linker connecting the AMP region and the pheromone for targeted bacteria. One possibility to test is combining the S. mutants UA 159 mature pheromone GLDWWSL [272,273] with short but powerful broad-spectrum antimicrobial peptide RRLFRRILRWL [156]. With the same GGG linker, we designed specifically targeted chimeric AMP: GLDWWSLGGGRRLFRRILRWL, which is considerably shorter (21 amino acid residues) and cheaper to synthesize than the C16G2 peptide (35 amino acid residues). It has a very high hydrophobic moment for an amphipathic helix in the second half of its sequence. The hemolytic activity prediction for that peptide decreased to an acceptable magainin 2 probability (0.823). The predicted toxicity score is substantially lower (−1.52).

For gangrene-causing *Streptococci* sp., some other Streptococci-specific pheromones can be helpful, either alone [274], or when combined with a broad-spectrum AMP. For instance, it may be interesting to test the SilCR competence-stimulating peptide DIFKLVIDHISMKARKK linked with GGG triplet to RRLFRRILRWL or KNLRIIRKGIHIIKKY AMP when *Streptococcus pyogenes* or *Streptococcus dysgalactiae* is detected in necrotizing tissue. In the case of *Streptococcus oralis*, implicated in throat infection or dental plaque formation, the pheromone choice can be DWRISETIRNLIFPRKK. For multi-drug-resistant Streptococcus strains, it would be advantageous to have an alternative option of antibiotics. The few examples we described for chimeric-targeted AMPs are only a minuscule portion of all possibilities. Still, the critical point here is that we can perform the rational design of promising chimeric peptides in silico before testing in the laboratory.

### 4.7. The Optimization of Multifunctional Constructs

Table 5 peptides 31–52 represent in silico attempts to answer different questions about the design of multifunctional peptides. A rational approach toward better anti-inflammatory activity increased the overall score of MFC (peptide 3) fused with short pexiganan analog (peptide 35) enough to classify it among the best 20 multifunctional peptide constructs (seventh). The same approach was successful with the PR-35 analog (peptide 45), the 13th peptide in the overall rank (Table 6). The parent peptide for the PR-35 analog is the antimicrobial PR-39 cathelicidin from the pig (the P80054 UniProt entry). Interestingly, all seven automatic substitutions replaced prolines to increase the predicted anti-inflammatory activity without decreasing the potential for CPP and most other PR-39 and PR-35 functionalities (compare peptides 41, 44, and 45 from Table 5).

Cecropin-magainin-2 hybrid peptide 39 (dubbed P18 by Shin et al. [185]) is the opposite example when suggested amino acid substitutions by the Anti-inflammatory server by Gupta et al. [35] produced its analog (peptide 46) with a high probability for hemolytic activity and no toxicity decrease. Substitution of central Pro residues with Leu eliminated low hemolytic activity predicted and observed for P18. However, substitutions suggested by the ToxinPred server by Gupta et al. [37,38] and the HeliQuest server by Gautier et al. [275] decreased the predicted hemolytic and toxic activity. In the optimized sequence KW**R**LFKKI-P-**R**FL**R**SA**RR**F (peptide 49 from Table 5), we selected substitutions that replaced all but the first cationic residue with Arg. We rejected all substitutions for central proline residue to maintain the high selectivity [222]. The other five servers predicted better multifunctional activities for that highly amphipathic helical peptide CA-MA2-analog2, including its cell-penetrating ability.

The amphipathic peptide LKLLKKLLKKLLKLL-NH_2_ (peptide 40, named K6L9) does not look promising due to observed and predicted potent hemolytic activity [186]. Still, its good antimicrobial and anticancer properties [276] stimulated the search for non-hemolytic analogs. For helical peptides with a continuous hydrophobic face, the selectivity can be increased together with the reduction in the hemolytic activity by inserting charged or D-amino acid residues into that helix face [277,278]. The LKlLKkLlkKLLkLL-NH_2_ analog of K6L9, named D-K6L9, has five D-amino acid residues (lower case letters indicate D-amino acids). It does not show any hemolytic activity, and it is better protected from in vivo cleavage by proteases [186]. Another ingenious chemical modification is the introduction of the site-specific isopeptide bond switch in K6L9. One such peptide, Amp1EP9 [279], is a stable and non-toxic antimicrobial peptide with other possible beneficial functions, such as anticancer and cell-penetrating. Unfortunately, the servers used in this review work only for the proteinogenic amino acids interconnected with peptide bonds. We can, however, imitate the D-K6L9 peptide by Gly and Arg substitutions into sequence locations 3 and 8 (Gly substitutions) and 6, 9, and 13 (Arg substitutions). The resulting LKGLKRLGRKLLRLL-NH_2_ peptide has a considerably lower probability of hemolytic activity (0.153 instead of 0.907) with similar predictions for all other functionalities.

Like PR-39, pyrrhocoricin is also a proline-rich antibacterial peptide (peptide 42 from Table 5). That host defense peptide from insects is devoid of in vitro or in vivo toxicity and has confirmed low hemolytic activity [187,280] (probability of 0.004 according to the HAPPENN server). Akin to other proline-rich peptides, pyrrhocoricin can enter a cell’s cytoplasm and exhibits multiple functions [280]. A recent finding is that the PRP repeat from pyrrhocoricin blocks the exit tunnel of 70S bacterial ribosome, which is essential for synthesizing all proteins [281,282]. Together with its cell-penetrating ability, this would explain the very high selectivity index and nanomolar concentration of pyrrhocoricin, which is enough to kill *E. coli* D22 and *Agrobacterium tumefaciens* [187]. It may be possible to broaden and strengthen the activity spectrum of pyrrhocoricin by fusing it with asparagutin (see Table 5 results for peptide 43).

### 4.8. Antimicrobial Peptides with Anticancer Activity Fused to Cell-Penetrating Peptides

A common theme in research about cancer and multidrug-resistant bacteria is the toxic side effects of last-resort drugs and natural obstacles impeding them from reaching their targets. Multifunctional peptides have the potential to overcome both hindrances. Besides magainins, many other natural peptides have verified antimicrobial and anticancer activity. Antibacterial AMPs with anticancer activity (ACP) are often cytotoxic to healthy human cells, but some are highly potent against bacteria and cancer cells while harmless to normal mammalian cells. Hoskin and Ramamoorthy [1] introduced classifications based on two general modes of AMP anticancer activity and several structural features in their influential review.

The structure of BMAP peptides, cecropins, LL-37, hCAP-18, magainins, temporins, fowlicidins, gaegurins, aureins, citropins, brevinins, ranatuerins, melittins, and their analogs is predominantly amphipathic α-helical in the membrane environment. Melittins are cytotoxic to all cells. Defensins, lactoferricins, and tachyplexins form amphiphilic β-sheet structure, while Pro-Arg-rich cathelicidin PR-39 and pyrrhocoricin lack the secondary structure. Some ACPs have a cyclic structure usually formed by disulfide bonds. Gomesin, tachyplexin I, and defensins are well-known examples. Our DADP database of anuran defense peptides ([283]; http://split4.pmfst.hr/dadp/, accessed on 7 August 2022) contains 108 peptides with dual AMP and ACP functions.

Gaspar et al. [2] enlisted 18 primary sequences for peptides with published data about their anticancer activity toward solid and hematological tumors. They concluded that the remaining challenges are delivery to tumor cells and lowering toxicity profile against healthy cells. The review of Deslouches and Di [171] lists 18 representative AMPs exhibiting anticancer activity as promising targets for drug development. The ADP database version 3 ([284]; https://aps.unmc.edu/AP/, accessed on 7 August 2022) contains 266 AMPs with anticancer activity. That is close to 8% of all their entries for antimicrobial peptides (a total of 3425 peptides). A richer CAMP_R3_ database with more than ten thousand antimicrobial peptides contains even more ACPs. The CancerPPD database [285] encompasses more than 600 experimentally confirmed anticancer peptides. Felício et al. [3] concluded their review of dual AMP and ACP activities with a statement that at least 10 of these peptides can be approved for clinical applications during the next five years. Low selectivity, high production costs, and low resistance to proteolytic cleavage slowed down the progress in the drug delivery pipeline. Still, some peptide candidates exhibited cytotoxic activity and good selectivity against multidrug-resistant cancer cells.

A more recent review by Tornesello et al. [286] mentions only one natural dual-action peptide (AMP and ACP), which reached phase II of clinical trial steps for the melanoma target. It is the LL-37 peptide with the primary structure: LLGDFFRKSKEKIGKEFKRIVQRIKDFLRNLVPRTES.

The LL-37 is one of the best-known multifunctional peptides and the only cathelicidin expressed in humans. Nijnik and Hancock [287] enumerated 12 different experimentally confirmed functions for LL-37, including immune modulation, wound healing, and angiogenesis, besides its antimicrobial and inhibition of biofilm formation activity. They did not discuss early indications of its anticancer, antiviral, antifungal, DNA binding, and cell-penetrating activity. Two LL-37 weaknesses are its weak potential for cell penetration (probabilities 0.68 and 0.45 for, respectively, CPP activity and uptake efficiency according to the MLCPP server) and low therapeutic index between 3 to 5 due to its toxicity to eukaryotic cells at slightly higher concentrations [1]. The selectivity index measured by hemolysis and minimal inhibitory concentration for bacterial growth is about 20 [288]. Regarding anticancer activity, LL-37 suppresses tumorigenesis in gastric cancer, but there is a perplexing implication for LL-37 in promoting breast, ovarian, and lung cancers [289].

Efforts to minimize the cost of peptide synthesis identified the LL-37 central helical region as the most important for its antibacterial, antibiofilm, and antiviral activity [290]. The same author (Guangshun Wang) subsequently added glycine at the N-terminal of their peptide GF-17 with the primary structure FKRIVQRIKDFLRNLV, which retained some antimicrobial and anticancer activity. To make it more resistant to proteases and more potent against multidrug-resistant ESKAPE bacterial species, Wang et al. [291] substituted two L-isoleucines and one L-leucine with three D-leucines. They also introduced several chemical modifications to make it more hydrophobic [291]. In the most active stable version of the GF-17 peptide, these authors replaced both phenylalanines with biphenylalanines. Substitution of Phe for biphenylalanine residues increases peptide hydrophobicity and self-assembly propensity. The resulting GF-17 analog, named 17BIPHE2 by Wang et al. [291], was equally potent against the *S. aureus* USA300 MRSA strain and the Gram-negative multidrug-resistant strains (MIC = 3.1 μM) with considerably higher SI = 73 compared to its parent peptide LL-37.

In our studies on how peptide antibacterial performance changes between Gram-negative and Gram-positive species [292], we have seen that high selectivity is more difficult to achieve against Gram-positive species such as *Staphylococcus aureus*. One possible reason is that more active peptides against *S. aureus* strains are more hydrophobic and more toxic to human cells. This makes it challenging to find the best compromise between low toxicity to healthy human cells and high wide-spectrum potency against most pathogenic bacteria and cancer cell types. Nevertheless, the 17BIPHE2 peptide exhibits 16 times better performance PE = SI/MIC than pexiganan’s performance against *S. aureus* strains (see reference [292] for antibacterial performance definition and estimates). Still shorter LL-37 dodecapeptide with one D-Leu residue in its primary structure KRIVK*L*ILKWLR, named KR-12-a5(6-DL) by Kim et al. [293], had a mean MIC = 3.4 μM, and SI = 61.2 (D-Leu at 6th location is in italic font).

In our experience, the majority of natural or designed peptide antibiotics with an excellent performance against a broad spectrum of Gram-negative and Gram-positive bacteria (including some multidrug clinical isolates) are likely to exhibit some degree of selective anticancer activity too. Good examples are the peptides we designed and named trichoplaxin-2a, pexiganan-L18, flexampin, zyk-1, adepantin-1a, and mapegin [88]. Their respective sequences are: RHHWRRYARIGFRAVRTVIGK (T2R1), GIGKFLKKAKKFGKAFVLILKK (PEXA), GIKKWVKGVAKGVAKDLAKKIL (FLEX), GIGREIIKKIIKKIGKKIGRII (ZYK1), GIKKAVGKALKGLKGLLKALGES (A1A), and KIGKKILKALKGALKELA (MAPA). For prostate cancer PC-3 cells, the IC50 concentrations ranged from 1.5 (Zyk-1) to 12 μM (A1A), which is 40 to 5 times stronger anticancer activity compared to the Polybia-MP1 anticancer peptide IDWKKLLDAAKQIL-NH2 [88,294].

There are other examples when experimental confirmations exist for the conjugates to target cancer cells or their organelles [146,147,158,160,295]. Conjugates with reversed optimal penetratin (peptides 17, 18, 20, and 21 from Table 2) belong to the same category. Their cancer-homing C-terminals are tLyP-1 peptides or their analogs (see peptide 1 from Table 5). Such peptides can be the artificial death signal for malignant mitochondria and tumors. The associated probability for hemolytic activity is negligible (see the HAPPENN server results from Table 2). Thus, therapeutic applications are possible for nontoxic or weakly toxic anticancer peptide conjugates with tLyP-1, even when one of the two servers we used does not predict anticancer activity.

A particular class of anticancer peptides can elicit tumor eradication through cytotoxic T-cell responses. For instance, cancer vaccination is performed with telomerase peptide EARPALLTSRLRFIPK named GV1001 [296]. The peptide can internalize into the cell cytoplasm [154]. Uptake efficiency prediction is boosted from low to high when the GG linker is introduced, and asparagutin is attached to construct the hybrid peptide 24 from Table 3.

Transforming dual-function (antimicrobial and anticancer) into a multiple-function peptide is easy in silico. One example is the asparagutin–adepantin hybrid sequence (peptide 18 from Table 4), which ranks 19th without substitutions (see overall rank from Table 6). This would not be possible if the conjugate did not excel at all six predicted activities in combination with low toxicity. One amino acid substitution in the adepantin 1A (Gly15 replacement with Leu15) increased the anti-inflammatory activity score from 1.36 to 1.62, according to the AntiInflam server. Still, the overall score decreased from 19th to 21st (see peptide 51 in Table 4 and Table 6). It illustrates how easily optimizing anti-inflammatory activity can increase hemolytic activity and decrease other beneficial functions.

### 4.9. Design Examples for Low Toxicity and Multiple Activities

The design for common antimicrobial, anticancer, and cell-penetrating ability can start with known AMP to which CPP is fused to increase the cell-penetrating efficiency of a hybrid peptide. It can also begin with known CPP by introducing amino acid substitutions to widen its activity spectrum. Let us first describe how we achieved the goal of in vitro antibacterial and anticancer activity for a modified CPP named mapegin [88]. Its parent CPP is well-known MAP sequence KLALKLALKALKAALKLA [166]. Rational design by Juretić et al. [88] resulted in the mapegin sequence K**IGK**K**I**LKALK**G**ALK**E**LA (named MAPA). It differs from the MAP sequence in highlighted and underlined amino acid residues I2, G3, K4, I6, G12, and E16, which increased flexibility (due to two glycines) but did not decrease the high amphipathicity feature of the parent peptide. We confirmed the predicted decrease in hemolytic activity and good antibacterial and anticancer activity. Minimal inhibitory concentrations of mapegin against *E. coli* and *S. aureus* bacteria (including drug-resistant strains) ranged from 0.5 to 8 μM, while IC50 against PC-3 prostate cancer cells was 8 μM [88].

Selectivity (toxicity absence) was not so good. For healthy human fibroblasts, the therapeutic index was about three. Regarding the hemolysis of human erythrocytes, the selectivity index was variable for different bacterial strains but more often on the low side. For *E. coli* and *S. aureus* the SI range was 10 < SI < 40. The 50% hemolysis after mapegin application was reached already with the peptide concentration of 20 μM. It is still an improvement in the hemolytic activity of the parent peptide (MAP), which is toxic to red blood cells. Moreover, mapegin is at least two times stronger antibacterial compound than MAP. The probability of hemolytic activity is low for mapegin, according to the HAPPENN server (0.079). Predicted cell-penetrating, antifungal, and anti-inflammatory activity of the mapegin await experimental confirmation. The cell-penetrating activity is expected to decrease due to six amino acid substitutions introduced into already excellent MAP CPP.

If we want to regain an excellent CPP function, the mapegin can be fused to some known CPP, such as the TAT peptide. We formed hybrid peptides mapegin–TAT (T3-48), mapegin–TAT analog1 optimized for higher anti-inflammatory activity (T3-49), and mapegin–TAT analog2 optimized for lower toxicity (T3-50). These are peptides 48–50 from Table 3. Their good overall rank (27th, 30th, and 11th, Table 6) makes all of them interesting for various applications. The disadvantage of hybrid peptides is their longer length and the increased cost to synthesize them.

We performed the rational design to obtain wide-spectrum antibacterial compounds before any tests on cancer cell lines [88]. Some dual-function peptides (PEXA, FLEX, ZYK1, A1A, and T2R1) are as good initial choices for creating hybrid peptides as the mapegin (see predictions for peptides 17–21, 35, 46–49, and 51 from Table 4). Observed MIC concentration values against *E. coli* ATCC 25922 and *S. aureus* ATCC 29213 were around one micromolar for all these peptides. The activity and the therapeutic index TI were surprisingly good against human prostate PC-3 cancer cells. After comparing peptide toxicity toward healthy human fibroblasts, we observed that the TI range was from about 3 (for mapegin and pexiganan-L18) to 10 (trichoplaxin-2a) [88]. Thus, for these six peptides, the therapeutic index tested on PC-3 cancer cells is not as high as the selectivity index for bacteria, which ranges from about 10 to more than 1000. Nevertheless, it is better than the TI for the anticancer peptide MP1 [294,297], which we used as a control. Since MP1 exhibits a moderate anticancer activity on tumor cell lines (around IC50 = 50 μM), our peptide antibiotics also have considerably better activity against cancer cells. There are, of course, other examples of how one can modify CPP or AMP templates for designing their anticancer or multifunctional analogs [1,3,5,6,12,166,298,299,300,301].

Our choice of online servers, mACPpred and ACPred, for anticancer activity is subjective and subject to flaws. There are some contradictory predictions for the anticancer activity (peptides 3–6, 14, and 15 from Table 2; peptides 6, 8, 28, and 29 from Table 3; peptide 41 from Table 4; and peptide 36 from Table 5). The reader can notice that the ACPred server frequently gives the ACP probability of around 0.98. This would be difficult to falsify in experiments because there is always the possibility that the peptide is active against a particular cancer cell line but inactive against other malignant cell types.

The lack of toxicity for proliferating human cells is questionable if a permanent blockage occurs for selected transcription sites in human DNA. On the other hand, a surrogate peptide that inhibits DNA binding of transcription factors needed for cancer cell proliferation may be useful in cancer treatments. It would be a welcome outcome for our hybrid peptides to directly prove their worth as anticancer peptides. Novel short CPP can serve as penetratin to import anticancer cargo drugs to desired internal targets in tumor cells. There are many other DNA/RNA-binding cryptides that can be used directly or in a modified form to increase libraries of multifunctional peptide assets. All transcription factors (TF) are prospective parent proteins for such peptides.

## 5. Summary Comments about Peptide Constructs

All the 20 best peptides (1st to 20th in the overall rank) have a high probability of intrinsic disorder throughout their length (see Table 6 legend). Due to their plasticity, there is no conflict with assuming a partially ordered structure in a suitable microenvironment. They often obtain an amphipathic secondary structure consisting of two arms with a flexible linker between them (α-helix or β-strand-hinge-α-helix or β-strand) when bound to an anionic membrane surface. After cell penetration and interaction with internal macromolecules, the peptides can change their conformation again. There is a high probability of forming DNA or RNA contacts, but it differs in the extent and sequence location among different peptides and their segments. For the best 20 peptides, the predicted binding sites with nucleic acids encompass 41% (sixth) to 100% (first and third) of their length (see Table 6 legend). Predicted protein binding residues make up from 10% to 70% of their length.

The spectrum of the most disordered and malleable structures adapting the conformation to different targets is not reserved for the listed Table 6 sequences of two-arm peptides. From the remaining nine Table 6 peptides and other Table 2, Table 3, Table 4 and Table 5 sequences, there are also examples when all of their residues are predicted with disordered conformation and high binding probability to nucleic acids. This is the case for the 22nd peptide, which is the conjugate of reversed optimal penetratin analog with the tLyp-1 analog (see peptide 21 Table 2), and the T2R3G3 construct with an overall score of 0.7981 (see peptide 34 from Table 3). The T2R3G3 is a modified trichoplaxin 2 analog sequence after adding two N-terminal and three C-terminal residues. It is a highly amphipathic α-helix membrane-binding structure for its central 6–21 segment (SPLIT algorithm prediction). The only outstanding feature of the first peptide (temporin analog fused to asparagutin analog) is its absence of predicted protein-binding contacts and the perfect separation between DNA-binding (1–11) and RNA-binding segment (residues 12–25).

We verified that with different scoring methods, temporin-CPP hybrids with a central bend interrupting helical structure are still top-ranking multifunctional peptides. Glycine, as a single or double linker in the central position, allows for a greater freedom of movement and better exploration of targets for the hybrid peptides. Increased flexibility contributes to better selectivity and lesser toxicity of hybrid peptides containing such a linker. Higher selectivity is the outcome for some of the designed peptides when central proline residue or proline doublet introduces the hinge between bioactive and cell-penetrating peptide segments.

Temporins were described and named by Simmaco et al. [302] as the smallest natural antibacterial peptides known at that time. They were first found from the skin secretion of *Rana esculenta* [303] and *Rana temporaria* [302], amphibian species widely distributed in Western and Central Europe. The top-listed in silico-designed candidates (Table 6) are certain temporin analogs fused to the RRWKIVVIRWRR, RRWFRRRRRR, or KRKRWHW cell-penetrating peptides. Natural temporins are amidated at their C-terminal, have a low net charge (from −1 to +3), and have a short length of between 8 and 17 amino acid residues [304,305]. Typically, they exhibit an amphipathic α-helical conformation in a nonpolar environment. Low toxicity to healthy mammalian cells, low cost for their synthesis, and multifunctional activity against bacteria, viruses, filamentous fungi, yeasts, protozoa, and cancer cells are well-known advantages of some natural temporins [304]. Temporin L, with the highest net charge (+3), has the broadest activity spectrum [306].

The therapeutically promising ability of temporins is that they do not harm macrophages at concentrations lethal to these cells’ intracellular parasites [304]. Anti-protozoa activity was not considered in our review, but neither were the anti-endotoxin, chemotactic, synergistic, and anti-biofilm formation activities attributed to temporins [307,308]. Of special interest are anticancer, antiviral, and fungicidal abilities of some temporins [304,309,310].

Synthetic analogs are often better than their “parent” peptides for desired activity. Shang et al. [112,311] examined highly charged analogs of temporin 1CEb starting from its sequence ILPILSLIGGLLGK-NH_2_ [162]. One of these analogs with six lysines and the sequence IKKIVSKIKKLLK-NH_2_ was named L-K6V1 [112]. It forms considerably less hydrophobic and more amphipathic helix in a membrane environment. Regarding their functionality spectrum, the analog gained better cell-penetrating and antimicrobial ability while losing its hemolytic activity (compare peptides 39 and 40 from Table 3). These improvements are much more apparent in experimental validations [112]. The L-K6V1 peptide (peptide 40, Table 3) still does not enter among the 20 best peptides from Table 2, Table 3, Table 4 and Table 5 (Table 6). It, however, served in turn as the “parent “peptide for fusing it with short and powerful CPP, such as the KW peptide (peptide 1, Table 4) or asparagutin (peptide 3, Table 3).

The broadest spectrum of best predictions is with the asparagutin analog RRWFR**S**RRRR, Gly-Gly linker, and L-K6V1 analogs. One of these sequences, the temporin-asparagutin analog 3 (peptide 37, Table 3) with the sequence **V**KKIVSKI**R**KLLK-GG-RRWFR**S**RRRR, ranked as the best one. The preliminary score (when toxic and hemolytic activity is not considered) and the overall score (when low toxicity is also considered in the overall mean score) agree on the highest ranking for that hybrid peptide.

Other temporin-asparagutin analogs with the G, GG, GGEPPKG, or GGGPPKG linker (Table 4, peptide 39; Table 3 peptide 36; Table 4, peptides 38 and 30; Table 3 peptide 9; Table 4 peptide 37; Table 3, peptide 35) ranked 2nd to 5th, 8th, 9th, and 14th, respectively, in the overall multifunctional score. The TA peptide 9 from Table 3 is already predicted with potent anti-inflammatory activity without needing any amino acid substitution. Sequences 30 from Table 4 (5th) and 48 from Table 5 (10th) are the shortest temporin-CPP conjugates with only 22 residues. To construct the 10th best peptide (peptide 48, Table 5), we used the novel P9 CPP carrier, RRRRRWWPP [188], as the reversed version (revP9) and added it to the C-terminal of L-K6V1 temporin [112]. One Pro residue remained near the central position after optimizing a hybrid peptide with the AntiInflam server. These nine temporin analogs are predicted with a nearly perfect score for antiviral activity. All of them enter among the 15 multifunctional peptides with the best overall score. The design of the 17th best peptide consisted in adding the N-terminal part of the first best peptide (**V**KKIVSKI**R**KLLKGG) to the CPP construct RRWKIVVIRWRR without any additional optimization. Among many possible applications, we can mention treating skin ulcers caused by the herpes virus. In any case, it is encouraging that in silico search for sequences with the best combination of multifunctional activities, intracellular targeting, and low toxicity zeroed on the class of temporin–CPP hybrids as 60% of the 15 best and 50% of the 20 best peptides. In contrast, ten temporin construct “winners” make up only about 6% of all peptides (176) we considered.

The second class of predicted top performers encompasses optimized penetratins and their analogs fused to the tumor-homing peptide tLyP-1. Optimal penetratin sequence GKRIGKKWKPRRRRFWRK with 18 residues (Table 2, peptide 22) ranks 31st among the best multifunctional peptides. We used the reversed optimal penetratin [56] as the parent peptide. The design consisted in increasing its alpha hydrophobic moment and applying several methods for improving its therapeutic index: locating the proline in the sequence middle, forming a hydrophobic sector interrupted with a charged residue, and introducing the small GXXXG motif at its N-terminal for stimulating peptides association in membrane environment [312]. We removed two C-terminal residues from the parent sequence KKWKPRRRRFWRKKR and added the pentapeptide GKRIG to its N-terminal to achieve these goals. A different approach is additional optimization for better anti-inflammatory activity and adding the tumor-homing peptide tLyp-1 [146] or its analog CGAKRTK to the C-terminal. The overall rank increased for hybrids 20 and 21 from Table 2 (6th and 22nd).

Our multifunctional construct RCGNKRFRWHW (peptide 3, Table 5) was useful when conjugated with the pexiganan analog optimized with two substitutions for better anti-inflammatory activity (T5-35). It ranked as the seventh best peptide. The predicted membrane-associated structure of MFC-PexS has a low profile of alpha and beta hydrophobic moments, distinguishing it from most other top-ranking peptides.

When fused mapegin and TAT CPP are optimized for low toxicity, the 11th peptide is obtained with 31 residues (Table 3, peptide 50). It has the lowest toxicity score of −1.81 and the highest reward score of 0.867 for the mean of low hemolytic probability and toxicity score. Any remaining confirmed activity (antiviral, antifungal, and anti-inflammatory) would be beneficial.

BMAP peptide analogs target mitochondria and cause apoptosis [174,313]. The most active peptide part (the 18 residues cathelicidin fragment from bovine) is fused to short CPP (the KW peptide). The top-scoring conjugates are peptide 33 from Table 4 (12th), and peptides 25 and 36 from Table 4 (16th and 18th). Optimizing peptide 25 from Table 4 for higher anti-inflammatory activity (with conservative substitution Leu for Ile) did not impair other beneficial functionalities of the peptide 33 sequence KRKRWHW-GGLRSLGRK**L**LRAWKKYG (Table 4).

Recently, experimentalists confirmed broad activity against enveloped viruses by the second bovine cathelicidin fragment with the sequence GRFKRFRKKFKKLFKKIS [179]. It was derived from BMAP-27 [314]. Its variant GRFKRFRKKFKKLFKKLS exhibited anti-parasitic activity [315]. We verified in silico that the hybrid peptide KRKRWHW-GRFKRFRKKFKKLFKKIS (peptide 52 from Table 4) is nontoxic for mammalian cells. Adding KW peptide conferred high multifunctional activities (32nd in the overall rank) without optimization. Thus, cathelicidin-CPP constructs are also promising lead compounds for multifunctionality.

We optimized only the best peptide candidates for higher anti-inflammatory activity. As a rule, we limited substitutions to three. One exception is the proline-arginine-rich peptide PR-35 (peptide 44 from Table 5). The optimized sequence RRR**V**RPPYLPR**V**RP**Q**PFFP**L**RL**LK**RI**S**PGFPPRFP has seven substituted residues (peptide 45 from Table 5). Its predicted toxicity to mammalian cells is low, and the overall rank is high (13th). There is, however, a decrease in expected cell-penetrating and anticancer activity compared to parent peptide PR-35.

Novispirin analogs also deserve several comments. The novispirin analog sequence KNLRIIRKGIHIIKKY (dubbed G2) lacks arginine at the fifth sequence location of novispirin-G10. It is used for anti-biofilm and anti-caries applications [269,270,316,317]. This was our starting peptide for creating and optimizing CPP chimeras. With KW CPP linked via Gly doublet after the G2 peptide, the optimization for lower toxicity resulted in the sequence KNLRI**F**RKGIHI**H**KKY-GG-KRKRWHW (T4-50), which scored 20th in the overall rank.

Intriguingly, 11 out of 20 best multifunctional peptides exhibit anticancer and antiviral probability close to 1.0 (>0.95, see Table 6 results from columns 5 and 6 highlighted in the gray background). A common feature of cancer phenotype and cell transformation into the viral factory is intensive bioenergetics [227], which is likely to be inhibited by antimicrobial peptides, such as temporin, BMAP, adepantin-1, and trichoplaxin-2 analogs.

## 6. Conclusions

Nature endowed host defense peptides with multifaceted activity. Natural AMPs with CPP activity, or CPP fragments, can interact with multiple sites of bacterial or fungal cells. There are hundreds of internal protein targets for penetratin, lactoferricin B, and PR-39, to name just a few well-known peptides explored with the protein microarray technique [318,319,320]. Thus, we should not constrain rational design to the “magic bullet“ goal. Some short synthetic CPP, such as Sub 5 [189] (see last rows of Table 5), have remarkably diverse internal protein targets [321]. Multiple targeting and rapid action minimize the chance of resistance development in targeted microorganisms or cancer cells. Marketed single-target drugs are frequently unable to reach internal targets and are prone to mistargeting with associated side effects. Fast-evolving microbes or malignant cells quickly develop resistance to such drugs. Deleterious effects then predominate benefits. However, targeting sequences conjugated to CPP offer a precision medicine tool for acting on well-protected organelles [322], intracellular pathogens, hijacked processes in pathological conditions, and foreign molecules in our cells.

Advanced prediction tools combined with expert design allow the construction of about 20 nontoxic CPP-hybrids with a high score for anti-inflammatory activity and a high probability (≥0.7) for the intrinsic disorder, cell-penetrating, antibacterial, antifungal, antiviral, and anticancer activity. Such flexible peptides with a high cationic charge often adapt the two arms structure after coming into contact with anionic molecules. For instance, an amphipathic helix-hinge-helix conformation can bridge different molecules and exhibit complex functionality. Designed peptides should pass easily through the plasma membrane in the eukaryotic cells. Their likely internal targets are respiring mitochondria, unprotected parts of nucleic acids, or negatively charged molecules in the cell wall and cytoplasmic membrane of bacterial cells. Multiple protein targets are also possible due to the wide range of predicted functions. In conclusion, the review is the argument for exploring wide-spectrum multifunctionality *in silico*, *in vitro*, and *in vivo*. Let us hope pharmaceutical companies and governmental regulations become less refractory to the multifunctional drug potential of cell-penetrating antimicrobial peptides and their conjugates.

## Figures and Tables

**Table 1:** Penetratin-like peptides within homeodomains.

Organism/ Common Name	Parent Protein or Gene/ GenBank or Uniprot Link	* Penetratin or Penetratin Analog Sequence	#Arg /#Lys/CPP Probability ^$^
*Drosophila melanogaster/fruit fly*	pAntp/P02833	RQIKIWFQNRRMKWKK	3/4/1.00
*Homo sapiens*/human	Hox-A5/P20719 PDX-1/P52945 HXD8/P13378 Hox-C12/ P31275	**RQIKIWFQNRRMKWKK** **R**H**IKIWFQNRRMKWKK** **RQ**V**KIWFQNRRMKWKK** Q**Q**V**KIWFQNRRMK**K**KR**	3/4/1.00 3/4/0.997 3/4/0.97 3/4/0.97
*Homo sapiens*/human	Pax-6/P26367 Pax-7/P23759 and Pax-3/P23760	AR**I**QV**WF**S**NRR**A**KW**RR ARVQV**WF**S**NRR**AR**W**R**K**	5/1/0.94 5/1/0.94
*Homo sapiens*/human	PITX2/D6RFI4	ARVRV**WF**K**NRR**A**KW**R**K**R	5/3/0.97
*Ciona intestinalis/*sea squirt tunicate	Pax3/7-like/NP_001071798.1	ARVQV**WF**S**NRR**A**KW**RR	5/1/0.94
*Acropora millepora/* stony coral	Pax-6/XP_029212196.2	AR**I**QV**WF**S**NRR**A**KW**R**K**	4/2/0.94
*Capitella teleta/* annelid worm	Ct-Pax3/7 (Pax6)/A1XC54, ABC68267.1	ARVQV**WF**S**NRR**AR**W**R**K**	5/1/0.94
*Nematostella vectensis/*sea anemone	PaxC homeodomain transcription factor/Q5IGV4	ARVQV**WF**S**NRR**A**KW**RR	5/1/0.94
*Mnemiopsis leidyi/* comb jelly	PRD10a homeobox trancription factor/ ADO22618.1	AR**I**QV**WFQNRR**A**KW**R**K**	4/2/0.93
*Amphimedon queenslandica/* sponge	Pax-6/XP_003387530.1	SRVQV**WFQNRR**A**KW**R**K**	4/2/0.93
*Trichoplax adhaerens*	PaxB/ACH57172.1	ARVQV**WF**S**NRR**A**KW**R**K**	4/2/0.92
*Ceratocystis platani/*fungi	Pax-6/KKF93291.1	AK**I**NN**WFQNRR**A**K**A**K**L	2/3/0.86
*Galerina marginata/Dykaria higher fungi*	Homeobox containing protein fragment/A0A067SZU8	AR**I**QV**WF**S**NRR**A**KW**RR	5/1/0.94
*Planoprotostelium fungivorum/amoeba*	Arf-GAP with homeobox domain/ A0A2P6NXG8	AR**I**QV**WF**S**NRR**A**KW**RR	5/1/0.94
*Monosiga brevicollis (Choanoflagellate)*	Mb_hbx2 homeobox-domain protein/ A9UP33	Q**QI**NN**WF**I**N**A**R**RRLLNR	4/0/0.76
*Capsaspora owczarzaki* amoebae * (Filasterea clade)*	CAOG_004648 Homeobox domain-containing protein/ A0A0D2VSA1	**R**V**I**R**IWFQNRR**A**K**QRR RRQKARRNQFWIRIVRR^§^	6/1/0.96 7/1/0.96
*Candida glabrata/*budding yeast	Homeobox containing protein PHO2/ Q6FKZ3	KNVR**IWFQNRR**A**K**VR**K** KNVR**IWFQNRR**A**K**VR**K**KGKL	4/3/0.95 4/5/0.95
*Hanseniaspora osmophila/* wine-making yeast	Regulatory protein PHO2 with homobox domain 1/A0A1E5RMZ3	T**Q**V**KIWFQNRRMKWK**R	3/3/0.94
*Acinetobacter baumannii/* Gram- bacteria	Homeobox domain-containing protein (partial)/ WP_139162288.1	**RQ**VAV**WFQNRR**AR**WK**T	4/1/0.87
*Klebsiella pneumoniae/* Gram- bacteria	Homeobox domain-containing protein WP_185963280.1	T**QIKIWFQNRR**A**K***D*HR	3/2/0.76
*Euryarchaeota archaeon*	RYE98021.1	RQVSV**WF**T**N**A**R**KRIWL	3/1/0.77
*Acanthamoeba polyphaga mimivirus/* giant virus	Putative homeobox protein/ AKI80488.1	**RQI**Q**IWFQNRR**C**K***D*R**K**	4/2/0.87
*Moumouvirus maliensis/*giant virus	Homeodomain containing protein/ QGR53678.1	K**QI**S**IWF**A**NRR**AY*D*ARK RKNGVKMTKVKKIRRSR^&^	3/2/0.63 4/5/0.94
*Megavirus chiliensis/*giant virus	Putative homeobox protein/ YP_004894234.1	**RQI**Q**IWFQNRR**AR*D*S**K**KNR	5/2/0.85
*Bandra megavirus/*	Homeobox/ AUV58136.1	**RQI**Q**IWFQNRR**AR*D*S**K**KIR	5/2/0.85
*Unclassified Mimivirus/* giant virus	Homeobox protein/ QZX43434.1	**RQI**Q**IWFQNRR**AR*D*SRKNR	6/1/0.86

* Bold font is for amino acid residues that are identical in type and sequence location to *Drosophila* pAntp penetratin; underlined residues are for extended penetratin analog at its C-terminal; All examined viruses and some Gram-negative bacteria have aspartate (D) highlighted with italic font instead of tryptophan (W) at the 14th sequence location. ^$^ #Arg/#Lys are the numbers of arginines and lysines in the sequence. The third number after the slash symbol is the cell-penetrating probability (CPP), according to www.thegleelab.org/MLCPP/ (accessed on 7 August 2022) server. **^§^** Reversed amoebae penetratin (Filasterea clade) with added arginine. ^&^ Homeodomain motif upstream from penetratin analog is also predicted as the CPP.

## Data Availability

Data is contained within the article.
